# Supplementary material for: Patterns and determinants of incident cataract surgery in China from 2011 to 2015 using a nationally representative longitudinal database
Source: BMJ Open. 2023 Jun 21;13(6):e069702. doi: 10.1136/bmjopen-2022-069702 (PMC10314420; doi:10.1136/bmjopen-2022-069702)
Supplement: Author's manuscript [file bmjopen-2022-069702.draft_revisions.pdf]

BMJ Open

BMJ Open is committed to open peer review. As part of this commitment we make the peer review history of every article we publish publicly available.

When an article is published we post the peer reviewers' comments and the authors' responses online. We also post the versions of the paper that were used during peer review. These are the versions that the peer review comments apply to.

The versions of the paper that follow are the versions that were submitted during the peer review process. They are not the versions of record or the final published versions. They should not be cited or distributed as the published version of this manuscript.

BMJ Open is an open access journal and the full, final, typeset and author-corrected version of record of the manuscript is available on our site with no access controls, subscription charges or pay-per-view fees (http://bmjopen.bmj.com).

If you have any questions on BMJ Open's open peer review process please email info.bmjopen@bmj.com

Patterns and Determinants of Incident Cataract Surgery in China from 2011 to 2015 Using a Nationally-representative Longitudinal Database

Journal:	*BMJ Open*
Manuscript ID	bmjopen-2022-069702
Article Type:	Original research
Date Submitted by the Author:	05-Dec-2022

Complete List of Authors:	Jan, Catherine; The University of Melbourne Department of Surgery in Ophthalmology, Ophthalmology<br>Xin, Jin; Peking University; Peking University<br>Dong, Yanhui; Peking University<br>Butt, Thomas; University College London<br>Chang, Robert; Stanford University, Ophthalmology<br>Keay, Lisa; University of New South Wales, School of Optometry and Vision Science<br>He, Mingguang; Sun Yat-Sen University Zhongshan Ophthalmic Center; Royal Victorian Eye and Ear Hospital, Centre for Eye Research Australia<br>Friedman, David; Harvard University, Ophthalmology<br>Congdon, Nathan; Queen's University Belfast

Keywords:	EPIDEMIOLOGY, Cataract and refractive surgery < OPHTHALMOLOGY, Ophthalmology < SURGERY

SCHOLARONE™
Manuscripts

I, the Submitting Author has the right to grant and does grant on behalf of all authors of the Work (as defined in the below author licence), an exclusive licence and/or a non-exclusive licence for contributions from authors who are: i) UK Crown employees; ii) where BMJ has agreed a CC-BY licence shall apply, and/or iii) in accordance with the terms applicable for US Federal Government officers or employees acting as part of their official duties; on a worldwide, perpetual, irrevocable, royalty-free basis to BMJ Publishing Group Ltd ("BMJ") its licensees and where the relevant Journal is co-owned by BMJ to the co-owners of the Journal, to publish the Work in this journal and any other BMJ products and to exploit all rights, as set out in our licence.

The Submitting Author accepts and understands that any supply made under these terms is made by BMJ to the Submitting Author unless you are acting as an employee on behalf of your employer or a postgraduate student of an affiliated institution which is paying any applicable article publishing charge ("APC") for Open Access articles. Where the Submitting Author wishes to make the Work available on an Open Access basis (and intends to pay the relevant APC), the terms of reuse of such Open Access shall be governed by a Creative Commons licence – details of these licences and which Creative Commons licence will apply to this Work are set out in our licence referred to above.

Other than as permitted in any relevant BMJ Author's Self Archiving Policies, I confirm this Work has not been accepted for publication elsewhere, is not being considered for publication elsewhere and does not duplicate material already published. I confirm all authors consent to publication of this Work and authorise the granting of this licence.

For peer review only

Title: Patterns and Determinants of Incident Cataract Surgery in China from 2011 to 2015 Using a Nationally-representative Longitudinal Database

Catherine Jan,[1,2,3] Jin Xin,[4,5] Yanhui Dong,[6] Thomas Butt,[7] Robert T. Chang,[8] Lisa Keay,[9] Ming He,[2,3] David S. Friedman[10,11] and Nathan Congdon[12, 13, 14]

1. Lost Child's Vision Project, Sydney, Australia
2. Centre for Eye Research Australia, Ophthalmology, Department of Surgery, University of Melbourne, Melbourne, Victoria, Australia.
3. Department of Ophthalmology and Surgery, Faculty of Medicine, Dentistry & Health Sciences, University of Melbourne, Melbourne, Australia
4. Institute of Population Research, Peking University
5. Peking University Library, Peking University, Beijing, China
6. Institute of Child and Adolescent Health, School of Public Health, Peking University
7. UCL Institute of Ophthalmology, University College London, London, UK
8. Ophthalmology, Byers Eye Institution at Stanford, Stanford, California, United States
9. School of Optometry and Vision Science, University of New South Wales, Sydney, Australia
10. Department of Ophthalmology, Harvard Medical School, Boston, Massachusetts, USA
11. Massachusetts Eye and Ear Infirmary
12. State Key Laboratory of Ophthalmology, Zhongshan Ophthalmic Center, Sun Yat-sen University, Guangzhou, China
13. ORBIS International, New York, USA
14. TREE Centre, Centre for Public Health, Queen's University Belfast, Belfast, Northern Ireland

For peer review only - http://bmjopen.bmj.com/site/about/guidelines.xhtml

Corresponding author: Catherine Jan, Department of Ophthalmology and Surgery, Faculty of Medicine, Dentistry & Health Sciences, The University of Melbourne, Swanston St PARKVILLE VIC Australia 3052, cjan541@gmail.com

Key words: Cataract surgery, epidemiology, healthcare access, equity,

Word count: 2667

For peer review only

For peer review only - http://bmjopen.bmj.com/site/about/guidelines.xhtml

Abstract

Purpose
To identify subpopulations at greatest risk for low utilization of cataract surgery, and to investigate determinants of cataract surgery in Chinese adults.

Methods
This nationally-representative longitudinal study recorded self-reported incident cataract surgery among urban and rural Chinese persons aged 45 years and older, and measured biological, clinical, and socioeconomical characteristics at baseline and endline.

Results
Among 16,663 people enrolled in 2011, 13,705 (82.2%) attended follow-up in 2015. Among these, 167 (1.22%) reported incident cataract surgery. Those receiving surgery were significantly older (66.2+/-8.79 vs 58.3+/-9.18, p=<0.001), and more likely to report: illiteracy (44.9% vs 27.1%, p<0.001), poor baseline distance vision (49.7% vs 20.0%, p=<0.001), poor baseline near vision (37.1% vs 21.8%, p=<0.001), baseline visual impairment (15.6% vs 5.5%, p=<0.001), diabetes (12.0% vs 7.42% ,p=<0.05), and higher baseline depression scores (9.7 vs 8.4 on a scale of 0-30, p=<0.05).
In linear regression models, older age, worse distance vision, hypertension or diabetes, illiteracy, and lower depression score were all significantly associated with undergoing surgery. Results were similar in models including only persons aged 60 years and above, except that urban residence was also associated with surgery. When only those aged 60 years and above with poor vision were included, results were again the same, except that higher household expenditure was also associated with surgery.

Conclusions
In China, cataract surgical rates remain low, but it appears governmental and non-governmental programs have reduced income-related barriers to access.

Key messages

What is already known in this topic?

Cataract surgery data from China is important to population studies due to the sheer size of China's population. However, there have been no published studies of cataract surgery incidence using nationally-representative samples in China.

What this study adds?

This is first large population study of determinants of surgery uptake in China, providing complementary information, as well as providing cataract surgery incidence data using a nationally representative sample.

How this study might affect research, practice or policy?

National prevalence and incidence data across China are vital for effective policy formulation and resource allocation, which can be a useful source of reference for both local Chinese health ministries as well as international bodies such as the World Health Organisation.

Synopsis
In China, 4-year incident cataract surgery remains as low as 1.22% and there is room for improvement.

For peer review only

For peer review only - http://bmjopen.bmj.com/site/about/guidelines.xhtml

Introduction

Blindness is among the most feared health conditions, ranking only after cancer and AIDS, and vision is the most valued of all the senses.[1] Age-related cataract is the world's and China's leading cause of blindness.[2,3] Surgery is currently the only available treatment for cataract, and has been rated among the most cost-effective procedures in medicine by the World Health Organization (WHO).[4] Cataract surgery improves quality of life, increases activities of daily living, and results in higher household economic productivity.[5-7] With a rapidly ageing population in China, a significant increase in the burden of cataract is expected, highlighting the importance of efforts to address this burden.

Previous population-based, cross-sectional studies have reported the rates of prior cataract surgery in different regions of China,[8-12] with population prevalence ranging from 2.9% in the Beijing Eye Study to 4.4% in Liwan, southern China. Rural cataract surgical coverage (CSC, proportion of individuals with vision-impairing cataract who have received surgery) for those with vision of <6/60 rose from 35% (range 24-62%) in 2006[8] to 63% (range 43-84%) in 2014, though coverage varied widely between provinces and was generally lower in poorer, western areas.[13] However, CSC in China, particularly rural areas, is still lower than in less wealthy neighboring countries such as India (67.2%). National prevalence and incidence data across China are vital for effective policy formulation and resource allocation. However, there have been no published studies of cataract surgery incidence using nationally-representative samples in China [14, 15] and most reports are from high-income countries.[16-18] The multiple barriers to accessing cataract surgery in China include cost (direct out-of-pocket surgical expense as well as transportation and accommodation[19,20]), lack of knowledge about cataract and cataract surgery,[20,21] and concerns about the quality of local surgeons.[22] Prior work in this area has several shortcomings. Firstly, no previous study has used a nationally representative sample which is needed because in a large country like China, social economic status (SES) and other determinants of cataract surgery uptake may vary widely by region. Secondly, many existing studies[19,20,22] have used data from prior to 2009, when China announced systematic

For peer review only - http://bmjopen.bmj.com/site/about/guidelines.xhtml

health reforms. Access to cataract surgery in urban and rural areas has improved substantially due to universal coverage under the Urban Resident Medical Insurance (URMI) and New Cooperative Medical System (NCMS) schemes. Few recent publications using national data on cataract surgery have been published.[23] Finally, while evidence from low-and middle-income countries suggests that health decisions are usually made at the household level, most existing studies in China have included only patient-level data, mostly concerning clinical rather than socio-economic variables.[19]

To address these limitations, the present paper employed a national longitudinal survey conducted in 30 out of 31 mainland Chinese provinces (except Tibet, though the study did include one Tibetan county), representative of the adult population 45 years and older, in order to understand patterns of cataract surgery incidence across China between 2011 and 2015. The aim was to identify subpopulations at greatest risk for low utilization, and to investigate the clinical, biological, and socio-economic determinants of and barriers to cataract surgery in adults aged 45 years and above in urban and rural China between 2011 and 2015.

Methods:

Study population

The China Health and Retirement Longitudinal Study (CHARLS) is a nationally-representative longitudinal survey among Chinese persons aged 45 years and older, that includes assessments of biological, social and economic conditions. We used data from the baseline study conducted between July and September 2011 ("Wave 1") and a follow up conducted (both surveys conducted in person) between July and September 2015 ("Wave 3"). Main respondents and spouses in the baseline survey are followed throughout the life of CHARLS, or until they die.[24] Detailed information on the methodology of CHARLS has been described elsewhere.[24] In brief, 17,708 people aged 45 years and older participated in the baseline study in 2011. Among them, 16,663 (94.1%) were eligible for data collection, 107 (0.604%) did not provide information on cataract surgery, 457 (2.58%) were aged

For peer review only - http://bmjopen.bmj.com/site/about/guidelines.xhtml

below 45 years, and 481 (2.72%) had already undergone cataract surgery before the baseline and were excluded from our sample. Among those eligible at baseline who provided information on cataract surgery in 2011 (n=16991, 96.0% of the total), 13705 (82.2%) attended the four-year follow-up in 2015. The protocols of the follow-up for the variables used in this paper followed those of the baseline study.

Samples were chosen through multistage probability sampling. In the first stage, 150 county-level units were randomly chosen with a probability-proportional-to-size (PPS) sampling technique from a sampling frame containing all county-level units with the exception of the Tibet Autonomous Region. The sample was stratified by region and within region by urban district or rural county and per capita statistics on gross domestic product (GDP). The final sample of 150 counties fell within 28 provinces. Our sample used the lowest level of government organization, consisting of administrative villages (*cun*) in rural areas and neighborhoods (*shequ* or *juweihui*) in urban areas, as primary sampling units (PSUs). We selected 3 PSUs within each county-level unit, using PPS sampling (more details are reported elsewhere).[24]

Variables

Cataract surgery incidence refers to the proportion of the cohort population aged 45 years and older who underwent cataract surgery in at least one eye between 2011 and 2015, among those who had not undergone cataract surgery in either eye at baseline. This was determined by participants' response to the question "Have you had cataract surgery before?", comparing answers between the previous and present round.

Age and gender were self-reported variables taken from the baseline 2011 survey. Distance and near vision were self-reported at baseline, using a scale of 1 to 5, with 1 being excellent and 4 and 5 being fair and poor respectively. Binary visual impairment was also self-reported.

For peer review only - http://bmjopen.bmj.com/site/about/guidelines.xhtml

Rural or urban place of residence was obtained from self-reported *Hukou*. *Hukou* is a Chinese internal passport system that is designed to regulate the influx of rural residents into urban areas, and migrants with rural *hukou* do not have equal access to health care compared to local urban residents. Hukou also limits the type of public health insurance the person has access to.

Three comorbidities linked with cataract and/or uptake of cataract surgery were extracted from CHARLS. Hypertension was defined as either self-report of a physician's diagnosis, or presence of objective biomarkers (systolic pressure >= 140 and/or diastolic pressure >= 90 mmHg) during direct examination. Diabetes was similarly defined by self-report of prior diagnosis by a doctor or the presence of a biomarker (HbA1c value ≥6.5%) on examination. Depression was measured using the Center for Epidemiologic Studies Depression Scale (CES-D-10)[25] with total score ranging from 0 (minimal symptoms) to 30 (most symptomatic).

Data were obtained from the National School of Development (China Centre for Economic Research) of Peking University. Secondary analysis of data from CHARLS did not require ethical approval. GDP data were extracted from the China Statistical Yearbook, and per capita household expenditure were derived from surveys of household expenditure divided by the number of household members. GDP in Chinese Yuan was converted to the US dollar using the exchange rate on 1st August, 2011, released by the center for foreign trade, Bank of China.

All data collected in the CHARLS are maintained at the National School of Development of Peking University, Beijing, China. The datasets are available from http://charls.pku.edu.cn/pages/data/111/zh-cn. html. The CHARLS was approved by the Ethical Review Committee of Peking University, and all participants gave written informed consent at the time of participation. This study used public data and therefore does not require additional ethics approval. The original CHARLS study from which the data were collected was carried out in Beijing and has obtained ethics approval from the Ethical Review Committee of Beijing University the ethical

For peer review only - http://bmjopen.bmj.com/site/about/guidelines.xhtml

approval number is IRB00001052-11015.

Patient and Public Involvement (PPI)

As this is an analysis of publicly available dataset, neither ethics nor PPI are applicable.

Statistical analysis

All statistical analyses were performed using Stata 13.0 (StataCorp., College Station, TX). The t-test was used to compare continuous variables, while Pearson X^2 or Fisher's exact tests were used for the comparison of categorical data. As a test to assess accuracy of household expenditure data from the sample as an index of income in the province, a scatterplot graph was made comparing household expenditure to provincial GFDP data. The 4-year incidence of cataract surgery stratified by age and gender were calculated. Age was defined in our analysis as the age at baseline. Univariate and multivariate logistic regression models were used to test for association with incident cataract surgery over the 4-year follow-up period. We ran three sets of regression analysis, one including the whole sample of eligible participants (i.e., all eligible people who were aged 45 years and above), one including those eligible participants aged 60 years and above (as visually-significant cataract is far more prevalent in this age range), and the third including those eligible participants aged 60 years and above who also reported fair or poor vision (the subset of participants at greatest risk for undergoing cataract surgery) . The variables in univariate models that had p value of less than 0.05 were deemed significant and were kept in the multivariate models. The incidence of cataract surgery in each province was affected by the age and gender structure of the population aged 45 years and over. Therefore, the provincial incidence of cataract surgery was adjusted for age and gender structure of the population. We applied sample weights to the cataract surgery incidence between 2011 and 2015. These weights were constructed from weights for the structure of age and gender from the baseline wave of the CHARLS study. A p value of <0.05 was defined as statistically significant.

For peer review only - http://bmjopen.bmj.com/site/about/guidelines.xhtml

Results:

Among the 16,663 eligible people aged 45 years and above (Table 1), 13,705 (82.2%) attended the year four follow-up. Among them, 13,538 (98.8%) did not report receiving surgery between 2011 and 2015, while 167 (1.22%) did. Those who received surgery were significantly older (66.2+/-8.79 vs 58.3+/-9.18, p=<0.001), and more likely to be illiterate (44.9% vs 27.1%, p<0.001), have self-reported poor distance vision at baseline (49.7% vs 20.0%, p=< 0.001), self-reported poor near vision at baseline (37.1% vs 21.8%, p=< 0.001), self-reported visual impairment at baseline (15.6% vs 5.5%, p=< 0.001), diabetes (12.0% vs 7.42% ,p=< 0.05), and higher depression scores at baseline (9.7 vs 8.4 on a scale of 0-30, p=< 0.05)

Table 1: Baseline characteristics of participants aged 45 and above who participated both the baseline and follow-up surveys.

**Baseline characteristic N=13705，>=45，drop missing cases**	**Cataract surgery N=167**	**No cataract surgery N=13538**
Age (years), mean (Std. Dev.)	66.17(8.79)	58.29(9.18)
Distance Vision status, n (%)		
Poor or fair	125 (75.9)	7782 (57)
Excellent, very good and good	32 (19.2)	4904 (36)
Hypertension, n (%)		
Yes	67 (40),	5238 (39)
No	76 (46)	6532 (48)
Diabetes, n (%)		
Yes	20 (12)	1004 (7.4)
No	102 (61)	8701 (64)

For peer review only - http://bmjopen.bmj.com/site/about/guidelines.xhtml

Male	71(42)	6556(48)
Female gender, n (%)	96(58)	6982(52)
Literacy, n (%)		
Literate	92(55)	9865(73)
Illiterate	75(45)	3673(27)
mean depression score, mean (SD)	9.70(6.53)	8.37(6.30)
Hukou, n (%)		
Rural	127（76.0）	10961（80）
Non-rural	39(23)	2484(18)
Log household per capita expenditure (USD), mean (SD)	4.57 (.94)	4.68(.88)
Health insurance, n (%)		
Yes	158 (95)	12664(94)
No	8 (5)	797(6)
Geographical location, n (%)		
Eastern region	52(31)	4704(35)
Central region	60(36)	4428(33)
Western region	55(33)	3306(34)
marital status		
married	131(78)	11066(82)
partner	6(4)	897(7)
separate	1(1)	60(0.4)
divorce	0	101(0.7)
Widowed	29(17)	1290(9.5)
never married	0	124(0.9)

For peer review only

Table 2 lists the 28 provinces for which data are available in the current study in order of per capita GDP by region and by province within region. There was a non-significant trend towards higher mean weekly household per capita expenditure in Eastern China (US$34.3) versus Central (US$20.5) and Western China (US$18.1). Mean provincial per capita weekly household expenditures as reported by study participants were highly correlated (R2=0.650, P=0.0018, regression correlation) with available data for per capita GDP by province (Figure 1).

Table 2: Characteristics of 28 Chinese provinces with regard to per capita gross domestic product (GDP), population and other characteristics, in 2011 (Arranged by region and province in order of reported per capita GDP)

Province name	Reported per capita GDP （USD, 2011）	Reported total population (thousands) (2011)	Reported population Density (persons/ square kilometre) (2011)	Number of participants in the current study （N=16663）	Mean weekly household per capita expenditure of study participants (n=16,663), 2011 (USD)	Mean household per capita expenditure, 2011, by geographic region (USD)
Eastern China						
Tianjin	13232.0	13,550	2636	149	44.9	34.3
Beijing	12680.0	20,190	1428	85	73.8	
Shanghai	12820.1	23,470	3702	58	58.6	
Jiangsu	9672.5	78,990	2013	834	38.8	
Zhejiang	9200.3	54,630	1741	659	48.0	
Guangdong	7889.4	105,050	2637	913	31.9	
Liaoning	7882.1	43,830	1712	510	24.3	

For peer review only - http://bmjopen.bmj.com/site/about/guidelines.xhtml

Fujian	7356.8	37,200	2306	483	36.5	
Shandong	7350.3	96,370	1389	1374	24.4	
Jilin	5972.1	27,490	2371	415	32.6	
Hebei	5274.8	72,410	2362	712	24.1	
Heilongjiang	5096.2	38,340	5146	312	39.5	
TOTAL	8702.2	50,960	2454	6504	39.8	
Central China						
Hubei	5310.2	57,580	1969	518	27.7	20.5
Shanxi	4869.2	35,930	2977	511	22.3	
Hunan	4639.8	65,960	2908	773	28.3	
Henan	4450.5	93,880	5124	1313	24.7	
Jiangxi	4060.6	44,880	4527	815	29.7	
Anhui	3984.4	59,680	2265	816	23.4	
TOTAL	4552.5	59,652	3295	4746	26.0	
Western China						
Inner Mongolia	9002.3	24,820	764	819	44.3	18.1
Chongqing	5357.2	29,190	1830	219	29.5	
Shaanxi	5196.4	37,430	5821	532	45.3	
Xinjiang	4672.0	22,090	4563	96	26.2	
Qinghai	4584.2	5680	2487	144	25.1	
Sichuan	4058.0	80,500	2782	1433	25.7	
Guangxi	3932.7	46450	1569	582	22.2	
Gansu	3042.7	25640	3824	402	24.8	
Yunnan	2991.5	46310	3811	997	21.7	
Guizhou	2548.6	34690	3502	189	23.9	

For peer review only - http://bmjopen.bmj.com/site/about/guidelines.xhtml

TOTAL	4538.6	35,280	3095	5413	28.9	

Among 7052 (42.3%) participants in the current study aged 60 and above, 126 indicated they had undergone cataract surgery during 2011 to 2015. Among 4522 (27.1%) participants aged 60 years and above and reporting "fair" or "poor" vision at the time of the most recent survey four years previously, data on cataract surgery were missing for 38 persons (0.840%), and a total of 98 (2.19% or 98/4484) indicated they had undergone cataract surgery, the 4-year incidence rate ranging from 2.43% (31/1401) in Central China, to 2.27% (35/1545) in the East to 2.08% (32/1538) in the West. (Table 3)

Table 3: Characteristics of persons aged 60 and over with self-reported vision of fair or poor in 2011 (N=4522) by region. Regions are listed in order of per capita GDP in 2011.

Economic region	Mean age (SD)	Women, n (%)	Literate, n (%)*	Mean depression score (95%CI) (SD)***	Rural (versus urban) residence, n (%)*	Mean household per capita expenditure, USD (SD)	Has only public health insurance, n (%)***	4-year cataract surgery incidence***, (%)
Eastern region	68.7 (0.181)	814 (52.7)	978 (63.3%)	9.18 （8.86-9.50） (0.164)	1,151 (74.6%)	24.7 (1.05)	1399 （90.6）	35/1,545 (2.27%)
Central region	67.8 (0.177)	705 (50.3%)	840 (60.0%)	10.1 （9.78-10.49） (0.179)	1,130 (80.7%)	19.7 (0.728)	1292 （92.2）	31/1,401 (2.43%)

For peer review only - http://bmjopen.bmj.com/site/about/guidelines.xhtml

Western region	68.4 (0.170)	791 (51.4%)	909 (59.1%)	10.9 （10.57-11.24） （0.17）	1,228 (79.8%)	21.2 (0.854)	1397 （90.8）	32/1,538 (2.08%)
TOTAL	68.34 (0.101)	2,327 (51.46%)	2,727/4484 (60.82%)	10.07 (9.88-10.23) (.099)	3509/4482 (78.29%)	21.8 (0.5)	4088/4422 (92.45%)	98/4484 (2.19%)

* P< 0.05, ** P < 0.01, *** P < 0.001, Chi square test for trend from highest to lowest cataract surgery incidence
****Data on cataract surgery was missing for 38 persons (0.840%)

For peer review only - http://bmjopen.bmj.com/site/about/guidelines.xhtml

In linear regression models of potential predictors of regional incidence of cataract surgery among persons aged 45 years and above at baseline, older age, worse vision, having hypertension or diabetes, being illiterate, and higher depression score were all significantly associated with increased surgery rates in the univariate model, and all these factors remained significant in multivariate model (Table 4). Results were similar in the multivariate regression model including only persons aged 60 years and above, except that urban hukou was also associated with higher incidence of surgery (Table 5). When only those aged 60 years and above with self-reported fair or poor vision were included, results in the multivariate model were again the same, except that higher household expenditure was also associated with higher incidence of surgery (Table 6).

Table 4: Linear regression model of potential predictors of the incidence of cataract surgery among all persons aged > =45 years (n= 16663, among whom 167 [1.00%] reported having surgery) across whole of China between 2011 and 2015 **(Bold type indicates values significant at P<0.05)**

Baseline Characteristic	Univariate model			Multivariate model		
	Beta coefficient	95% Confidence Interval	P-value	Beta coefficient	95% Confidence Interval	P-value
Age	**.094**	**-.0013 -.0009**	**<0.001**	**.080**	**.00098 .00106**	**<0.001**
Poor or Fair self-reported vision	**0.41**	**.005 .013**	**<0.001**	**.022**	**00485 .00625**	**<0.001**
Hypertension present	**.005**	**.001 .002**	**<0.001**	**-.018**	**-.0048 -.0035**	**<0.001**

For peer review only - http://bmjopen.bmj.com/site/about/guidelines.xhtml

(adjusted for age)						
Diabetes present (adjusted for age)	**.023**	**.008 .010**	**<0.001**	**.019**	**.0063 .0085**	**<0.001**
Female sex	.013	-.001 .007	0.129			
Literate	**-.0438**	**-.015 .007**	**<0.001**	**-.021**	**-.00614 -.00468**	**<0.001**
Mean depression score (Adjusted for age)	**.023**	**.0004 .0005**	**<0.001**	**.012**	**.00017 .00028**	**<0.001**
Rural (vs non-rural) hukou	-.014	-.0008 .0085	0.106			
Log household per capita expenditure	-.013	-.0038 .0006	0.146			
Reported having health insurance	.005	-.0054 .0102	0.550			

For peer review only

Table 5: Linear regression model of potential predictors of the regional incidence of cataract surgery among all persons aged > =60 years (n= 7052, among whom 126 [1.79%] reported having surgery) **(Bold type indicates values significant at P<0.05)**

Baseline Characteristic	Univariate model			Multivariate model*		
	Beta coefficient	95% Confidence Interval	P-value	Beta coefficient	95% Confidence Interval	P-value
Age	**0.060**	**.0007, .020**	**<0.001**	**.036**	**.00077 .00096**	**<0.001**
Poor or Fair self-reported vision	**.052**	**-.025 -.008**	**<0.001**	**.039**	**.01167 .01431**	**<0.001**
Hypertension present (adjusted for age)	**-.011**	**-.0043 -.0023**	**<0.001**	**-.023**	**-.00804 -.00567**	**<0.001**
Diabetes present (adjusted for age)	**.029**	**.0117 .0151**	**<0.001**	**.024**	**.00949 .01318**	**<0.001**
Female sex	.024	-.00005 .01499	0.068			
Literate	**-.032**	**-.0178 .0017**	**0.018**	**-.027**	**-.00957 -.00701**	**<0.001**
Mean depression score (Adjusted for age)	**.020**	**.000383 -.000536**	**<0.001**	**.023**	**.00044 .00062**	**<0.001**

For peer review only - http://bmjopen.bmj.com/site/about/guidelines.xhtml

Rural (vs non-rural) hukou	**-.030**	**-.0204 -.0015**	**0.024**	**-.043**	**-.01801 - .01489**	**<0.001**
Log household per capita expenditure	.011	-.00277 .00642	0.435			
Reported having health insurance	.001	-.016 .017	0.926			

For peer review only - http://bmjopen.bmj.com/site/about/guidelines.xhtml

Table 6: Linear regression model of potential predictors of the regional incidence of cataract surgery among all persons aged > =60 years and those who reported fair or poor vision (n= 4522) **(Bold type indicates values significant at P<0.05)**

Baseline Characteristic	Univariate model			Multivariate model*		
	Beta coefficient	95% Confidence Interval	P-value	Beta coefficient	95% Confidence Interval	P-value
Age	**.064**	**.0008227 .002492**	**<0.001**	**.035**	**.00076 .00101**	**<0.001**
Hypertension present (adjusted for age)	**-.014**	**-.00596 -.0032489**	**<0.001**	**-.039**	**-.01425 -.01100**	**<0.001**
Diabetes present (adjusted for age)	**.041**	**.0184 .0229**	**<0.001**	**.041**	**.01744 .02250**	**<0.001**
Female sex	.024	-.00278 .01839	0.148			
Literate	-.027	-.01995 .00171	0.099			
Mean depression score (Adjusted for age)	**.006**	**.0000485 .0002474**	**0.004**	**.027**	**.00053 .00078**	**0.004**
Rural (vs non-rural) hukou	**-.041**	**-.030558 -.003757**	**0.012**	**- .031**	**-.01515 -.01066**	**<0.001**

For peer review only

Log household per capita expenditure	**.036**	**.0001394 .012749**	**0.045**	**.012**	**.00130 .0032**	**<0.001**
Reported having health insurance	-.001	-.022986 .021785	0.958			

Discussion:

Between 2011 and 2015, the incidence of self-reported cataract surgery in the current study was relatively low (2.19%) among people aged 60 and above compared to earlier reports, which ranged from 2.9% in the Beijing Eye Study[10] to 4.4% in Liwan, southern China.[12] Various barriers to uptake of cataract surgery in China identified in prior studies include cost,[19,20] limited awareness of cataract and its treatment, [20,21] and uncertainty about local surgical quality.[22] More data are needed to help policy makers address these barriers. Previous studies have examined institutional predictors of higher surgical capacity at the institutional level (principally screening outreach activities). This is first large population study of determinants of surgery uptake in China, providing complementary information.

Our finding of lower surgical rates in more rural provinces is in agreement with results from prior population studies[8,13] which have also reported especially low rates of surgical uptake in rural settings. This finding indicates that this lower rate persists despite the rapid expansion of the NCMS rural health insurance program.[26,27]

For peer review only - http://bmjopen.bmj.com/site/about/guidelines.xhtml

With respect to individual-level variables, it was unsurprising that worse self-reported vision, as captured by three different variables, was significantly associated with greater uptake of surgery. As expected, older age was associated with elevated uptake of surgery.

Diabetes is well-established in population studies to be associated with higher risk of lens opacity, and so our observed higher prevalence of diabetes among those accepting surgery is not surprising. Cataract has also been linked with higher rates of mental health problems, including depression,[28] which we believe explains the observed higher rates of depression among those undergoing incident cataract surgery. An unexplained individual-level finding was a higher prevalence of self-reported illiteracy among those accepting incident cataract surgery. Poor education and lack of knowledge about cataract have generally been associated with low surgical uptake in previous population studies.[22-23] One possible speculation could be that illiterate people were more likely to do what the doctor recommended. Those with better education may have been more wary of using local services but could not afford to go to more distant locations for care. Alternatively, those with lower literacy may have other unmeasured factors that predispose to higher rates of cataract such as greater exposure to UV light.

Higher economic status (represented by higher weekly household per capita expenditure, which correlated strongly with per capita GDP, Fig 1) was associated with receiving cataract surgery. This result is expected, however, there have been several efforts in recent years that help provide surgery to those economically more disadvantaged. One is the roll-out of projects providing high-volume free surgery, such as the Million People Cataract Surgery Plan,[29] during the period covered in the current study. This has been supplemented by non-governmental organization (NGO)-supported cataract surgical programs by entities such as Lifeline Express,[30] and by the high penetration of the NCMS rural health insurance system, now covering some 98.9% of rural-dwellers[26] and substantially reducing out-of-pocket expenses associated with cataract surgery. We observed that the percentages of people covered by public health insurance did not vary significantly between poorer and richer regions. These programs taken together may have improved access in poor areas that reduced inequality in

For peer review only - http://bmjopen.bmj.com/site/about/guidelines.xhtml

cataract surgery access associated with income, but in the end, those with greater income were more likely to have cataract surgery.

The strengths of this study include a large nationally representative sample from most provinces of China, selected using standardized protocols. Study personnel were trained in standard fashion to ensure all procedures were carried out as described. Our findings provide a basis for comparison with other low- and middle-income countries and especially those undergoing rapid economic transitions. Limitations must be acknowledged as well. In the first place, the study did not measure objective visual acuity due to time and cost. Our main outcome, having undergone cataract surgery, depended on patient report for similar reasons, though evidence has suggested that self-reported cataract surgery is accurate and reliable.[31]

Conclusion:

Our study provides unique data on cataract surgical incidence in China. Our results suggest that rural and metropolitan both have access to cataract surgery. Surgical rates remain low, but reveal an encouraging phenomenon that a variety of governmental and non-governmental programs may have reduced income-related barriers to accessing cataract surgery in China.

Acknowledgement:

The authors would like to thank Professor Zhao Yaohui for her valuable comments and suggestions on this manuscript. We would also like to thank the CHARLS team for the data that enabled these analyses.

Author Contribution

For peer review only - http://bmjopen.bmj.com/site/about/guidelines.xhtml

Design: Jan and Congdon; Draft manuscript: Jan; Data analysis: Jan and Jin; Interpret data: Jan, Jin, Congdon, Friedman, Butt, Keay; Revise manuscript: Jin, Congdon, Butt, Dong, Friedman, Chang, Keay, He.

Competing interests: The authors have no competing interest.

Funding: N/A

Data sharing statement: All data collected in the CHARLS are maintained at the National School of Development of Peking University, Beijing, China. The datasets are available from http://charls.pku.edu.cn/pages/data/111/zh-cn. html.

Ethics approval statement: The CHARLS was approved by the Ethical Review Committee of Peking University, and all participants gave written informed consent at the time of participation. This study used public data and therefore does not require additional ethics approval. The original CHARLS study from which the data were collected was carried out in Beijing and has obtained ethics approval from the Ethical Review Committee of Beijing University the ethical approval number is IRB00001052-11015.

For peer review only - http://bmjopen.bmj.com/site/about/guidelines.xhtml

References

1. Enoch J, McDonald L, Jones L, Jones PR, Crabb DP. Evaluating whether sight is the most valued sense. *JAMA Ophthalmol* 2019; **137**(11): 1317-20.

2. Bourne RR, Stevens GA, White RA, et al. Causes of vision loss worldwide, 1990-2010: a systematic analysis. *Lancet Glob Health* 2013; **1**(6): e339-49.

3. Liang YB, Friedman DS, Wong TY, et al. Prevalence and causes of low vision and blindness in a rural Chinese adult population: the Handan Eye Study. *Ophthalmology* 2008; **115**(11): 1965-72.

4. Horton S, Gelband H, Jamison D, Levin C, Nugent R, Watkins D. Ranking 93 health interventions for low- and middle-income countries by cost-effectiveness. *PLoS One* 2017; **12**(8).

5. Lee BS, Munoz BE, West SK, Gower EW. Functional improvement after one-and two-eye cataract surgery in the Salisbury Eye Evaluation. *Ophthalmology* 2013; **120**(5): 949-55.

6. Wang JJ, Mitchell P, Smith W. Vision and low self-rated health: the Blue Mountains Eye Study. *Invest Ophthalmol Vis Sci*. 2000; **41**(1): 49-54.

7. Polack S, Kuper H, Mathenge W, Fletcher A, Foster A. Cataract visual impairment and quality of life in a Kenyan population. *Br J Ophthalmol*. 2007; **91**(7): 927-32.

8. Zhao J, Ellwein LB, Cui H, et al. Prevalence and outcomes of cataract surgery in rural China: the China nine-province survey. *Ophthalmology* 2010; **117**(11): 2120-8.

9. Lavanya R, Wong TY, Aung T, et al. Prevalence of cataract surgery and post-surgical visual outcomes in an urban Asian population: the Singapore Malay Eye Study. *Br J Ophthalmol*. 2009; **93**(3): 299-304.

10. Liu B, Xu L, Wang YX, Jonas JB. Prevalence of cataract surgery and postoperative visual outcome in Greater Beijing: the Beijing Eye Study. *Ophthalmology* 2009; **116**(7): 1322-31.

11. Li EY, Liu Y, Zhan X, et al. Prevalence of blindness and outcomes of cataract surgery in Hainan Province in South China. *Ophthalmology* 2013; **120**(11): 2176-83.

For peer review only

For peer review only - http://bmjopen.bmj.com/site/about/guidelines.xhtml

12. Huang W, Huang G, Wang D, Yin Q, Foster PJ, He M. Outcomes of cataract surgery in urban southern China: the Liwan Eye Study. *Invest Ophthalmol Vis Sci*. 2011; **52**(1): 16-20.
13. Zhao J, Xu X, Ellwein LB, et al. Cataract Surgical Coverage and Visual Acuity Outcomes in Rural China in 2014 and Comparisons With the 2006 China Nine-Province Survey. *Am J Ophthalmol* 2018; **193**: 62-70.
14. Zhang JS, Xu L, Wang YX, You QS, Da Wang J, Jonas JB. Five-year incidence of age-related cataract and cataract surgery in the adult population of greater Beijing: the Beijing Eye Study. *Ophthalmology* 2011; **118**(4): 711-8.
15. Huang W, Zheng Y, Wang L, et al. Five-year incidence and postoperative visual outcome of cataract surgery in urban southern China: the Liwan Eye Study *Invest Ophthalmol Vis Sci*. 2012; **53**(13): 7936-42.
16. Kanthan GL, Wang JJ, Rochtchina E, et al. Ten-year incidence of age-related cataract and cataract surgery in an older Australian population: the Blue Mountains Eye Study. *Ophthalmology* 2008; **115**(5): 808-14.
17. Klein BEK, Klein R, Lee KE. Incidence of age-related cataract over a 10-year interval: the Beaver Dam Eye Study. *Ophthalmology* 2002; **109**(11): 2052-7.
18. Koo E, Chang JR, Agrón E, et al. Ten-year incidence rates of age-related cataract in the Age-Related Eye Disease Study (AREDS): AREDS report no. 33. *Ophthalmic Epidemiol* 2013; **20**(2): 71-81.
19. Li Z, Song Z, Wu S, et al. Outcomes and barriers to uptake of cataract surgery in rural northern China: the Heilongjiang Eye Study. *Ophthalmic Epidemiol* 2014; **21**(3): 161-8.
20. Zhang M, Wu J, Li L, et al. Impact of cataract screening outreach in rural China. *Invest Ophthalmol Vis Sci* 2010; **51**(1): 110-4.
21. Yin Q, Hu A, Liang Y, et al. A two-site, population-based study of barriers to cataract surgery in rural China. *Invest Ophthalmol Vis Sci*. 2009; **50**(3): 1069-75.
22. Zhang M, Wu X, Li L, et al. Understanding barriers to cataract surgery among older persons in rural China through focus groups. *Ophthalmic Epidemiol* 2011; **18**(4): 179-86.

For peer review only - http://bmjopen.bmj.com/site/about/guidelines.xhtml

23. An L, Jan C, Feng J, Wang Z, Zhan L, Xu X. Inequity in access: Cataract surgery throughput of Chinese ophthalmologists from the China National Eye Care Capacity and Resource Survey. *Ophthalmic Epidemiol.* Accepted on October 7, 2019

24. Zhao Y, Hu Y, Smith JP, Strauss J, Yang G. Cohort profile: the China health and retirement longitudinal study (CHARLS). *Int J Epidemiol*. 2014; **43**(1): 61-8.

25. Lewinsohn PM, Seeley JR, Roberts RE, Allen NB. Center for Epidemiologic Studies Depression Scale (CES-D) as a screening instrument for depression among community-residing older adults. *Psychol Aging*. 1997 Jun;12(2):277.

26. Gu D, Yang X, Li X, Liang C, Zhong J, Feng N. Innovating new rural cooperative medical scheme (NCMS) for better patient satisfaction in rural China. Int J Env Res Pub Health. 2018 Sep;15(9):2007.

27. Drinkwater JJ, Davis WA, Davis TM. A systematic review of risk factors for cataract in type 2 diabetes. Diabetes/metabolism research and reviews. 2019 Jan;35(1):e3073.

28. Pellegrini M, Bernabei F, Schiavi C, Giannaccare G. Impact of cataract surgery on depression and cognitive function: Systematic review and meta-analysis. Clin Exper Ophthalmol. 2020 Mar 27.

29. Yan X, Guan C, Mueller A, Iezzi B, He M, Liang H, Meltzer M, Congdon NG. Outcomes and predicted impact on vision restoration of the China Million Cataract Surgeries Program. *Ophthalmic Epidemiol.* 2013;20:294-300.

30. Lifeline Express home page. http://www.lifeline-express.com/. Last visited 4 December 2020.

31. Bowie H, Congdon N, Lai H, West SK. Validity of a personal and family history of cataract and cataract surgery in genetic studies. *Invest Ophthalmol Clin Sci* 2003;44:2905-8.

For peer review only

Figure 1: Scatterplot and regression line showing the reported per capita gross domestic product of provinces (USD) against the mean household per capita expenditure (USD) of participants in our study from these provinces.

For peer review only

For peer review only - http://bmjopen.bmj.com/site/about/guidelines.xhtml

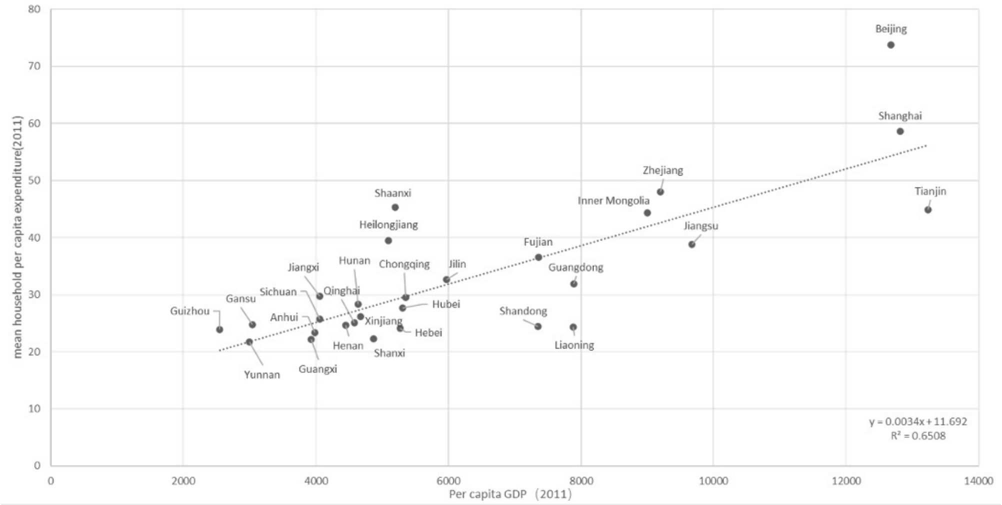

184x93mm (144 x 144 DPI)

STROBE Statement—checklist of items that should be included in reports of observational studies

		Item No	Recommendation
pages 1-3	**Title and abstract**	1	(*a*) Indicate the study's design with a commonly used term in the title or the abstract
			(*b*) Provide in the abstract an informative and balanced summary of what was done and what was found
	**Introduction**		
page 5	Background/rationale	2	Explain the scientific background and rationale for the investigation being reported
page 6	Objectives	3	State specific objectives, including any prespecified hypotheses
	**Methods**		
page 6	Study design	4	Present key elements of study design early in the paper
page 6-7	Setting	5	Describe the setting, locations, and relevant dates, including periods of recruitment, exposure, follow-up, and data collection
page 6-7	Participants	6	(*a*) *Cohort study*—Give the eligibility criteria, and the sources and methods of selection of participants. Describe methods of follow-up<br>*Case-control study*—Give the eligibility criteria, and the sources and methods of case ascertainment and control selection. Give the rationale for the choice of cases and controls<br>*Cross-sectional study*—Give the eligibility criteria, and the sources and methods of selection of participants
			(*b*) *Cohort study*—For matched studies, give matching criteria and number of exposed and unexposed<br>*Case-control study*—For matched studies, give matching criteria and the number of controls per case
page 7	Variables	7	Clearly define all outcomes, exposures, predictors, potential confounders, and effect modifiers. Give diagnostic criteria, if applicable
page 8	Data sources/ measurement	8*	For each variable of interest, give sources of data and details of methods of assessment (measurement). Describe comparability of assessment methods if there is more than one group
page 7	Bias	9	Describe any efforts to address potential sources of bias
page 7	Study size	10	Explain how the study size was arrived at
page 7-8	Quantitative variables	11	Explain how quantitative variables were handled in the analyses. If applicable, describe which groupings were chosen and why
page 9	Statistical methods	12	(*a*) Describe all statistical methods, including those used to control for confounding
			(*b*) Describe any methods used to examine subgroups and interactions
			(*c*) Explain how missing data were addressed
			(*d*) *Cohort study*—If applicable, explain how loss to follow-up was addressed<br>*Case-control study*—If applicable, explain how matching of cases and controls was addressed<br>*Cross-sectional study*—If applicable, describe analytical methods taking account of sampling strategy
			(*e*) Describe any sensitivity analyses

Continued on next page

For peer review only - http://bmjopen.bmj.com/site/about/guidelines.xhtml

	**Results**		
page 10	Participants	13*	(a) Report numbers of individuals at each stage of study—eg numbers potentially eligible, examined for eligibility, confirmed eligible, included in the study, completing follow-up, and analysed
			(b) Give reasons for non-participation at each stage
			(c) Consider use of a flow diagram
page 10	Descriptive data	14*	(a) Give characteristics of study participants (eg demographic, clinical, social) and information on exposures and potential confounders
			(b) Indicate number of participants with missing data for each variable of interest
			(c) *Cohort study*—Summarise follow-up time (eg, average and total amount)
page 12-20	Outcome data	15*	*Cohort study*—Report numbers of outcome events or summary measures over time
			Case-control study—Report numbers in each exposure category, or summary measures of exposure
			Cross-sectional study—Report numbers of outcome events or summary measures
page 12-20	Main results	16	(*a*) Give unadjusted estimates and, if applicable, confounder-adjusted estimates and their precision (eg, 95% confidence interval). Make clear which confounders were adjusted for and why they were included
			(*b*) Report category boundaries when continuous variables were categorized
			(*c*) If relevant, consider translating estimates of relative risk into absolute risk for a meaningful time period
	Other analyses	17	Report other analyses done—eg analyses of subgroups and interactions, and sensitivity analyses
	**Discussion**		
page 21	Key results	18	Summarise key results with reference to study objectives
page 23	Limitations	19	Discuss limitations of the study, taking into account sources of potential bias or imprecision. Discuss both direction and magnitude of any potential bias
page 23	Interpretation	20	Give a cautious overall interpretation of results considering objectives, limitations, multiplicity of analyses, results from similar studies, and other relevant evidence
	Generalisability	21	Discuss the generalisability (external validity) of the study results
	**Other information**		
	Funding	22	Give the source of funding and the role of the funders for the present study and, if applicable, for the original study on which the present article is based

*Give information separately for cases and controls in case-control studies and, if applicable, for exposed and unexposed groups in cohort and cross-sectional studies.

Note: An Explanation and Elaboration article discusses each checklist item and gives methodological background and published examples of transparent reporting. The STROBE checklist is best used in conjunction with this article (freely available on the Web sites of PLoS Medicine at http://www.plosmedicine.org/, Annals of Internal Medicine at http://www.annals.org/, and Epidemiology at http://www.epidem.com/). Information on the STROBE Initiative is available at www.strobe-statement.org.

For peer review only

BMJ Open

Patterns and Determinants of Incident Cataract Surgery in China from 2011 to 2015 Using a Nationally-representative Longitudinal Database

Journal:	*BMJ Open*
Manuscript ID	bmjopen-2022-069702.R1
Article Type:	Original research
Date Submitted by the Author:	04-Feb-2023
Complete List of Authors:	Jan, Catherine; The University of Melbourne Department of Surgery in Ophthalmology, Ophthalmology<br>Xin, Jin; Peking University; Peking University<br>Dong, Yanhui; Peking University<br>Butt, Thomas; University College London<br>Chang, Robert; Stanford University, Ophthalmology<br>Keay, Lisa; University of New South Wales, School of Optometry and Vision Science<br>He, Mingguang; Sun Yat-Sen University Zhongshan Ophthalmic Center; Royal Victorian Eye and Ear Hospital, Centre for Eye Research Australia<br>Friedman, David; Harvard University, Ophthalmology<br>Congdon, Nathan; Queen's University Belfast
**Primary Subject Heading**:	Public health
Secondary Subject Heading:	Ophthalmology
Keywords:	EPIDEMIOLOGY, Cataract and refractive surgery < OPHTHALMOLOGY, Ophthalmology < SURGERY

SCHOLARONE™
Manuscripts

I, the Submitting Author has the right to grant and does grant on behalf of all authors of the Work (as defined in the below author licence), an exclusive licence and/or a non-exclusive licence for contributions from authors who are: i) UK Crown employees; ii) where BMJ has agreed a CC-BY licence shall apply, and/or iii) in accordance with the terms applicable for US Federal Government officers or employees acting as part of their official duties; on a worldwide, perpetual, irrevocable, royalty-free basis to BMJ Publishing Group Ltd ("BMJ") its licensees and where the relevant Journal is co-owned by BMJ to the co-owners of the Journal, to publish the Work in this journal and any other BMJ products and to exploit all rights, as set out in our licence.

The Submitting Author accepts and understands that any supply made under these terms is made by BMJ to the Submitting Author unless you are acting as an employee on behalf of your employer or a postgraduate student of an affiliated institution which is paying any applicable article publishing charge ("APC") for Open Access articles. Where the Submitting Author wishes to make the Work available on an Open Access basis (and intends to pay the relevant APC), the terms of reuse of such Open Access shall be governed by a Creative Commons licence – details of these licences and which Creative Commons licence will apply to this Work are set out in our licence referred to above.

Other than as permitted in any relevant BMJ Author's Self Archiving Policies, I confirm this Work has not been accepted for publication elsewhere, is not being considered for publication elsewhere and does not duplicate material already published. I confirm all authors consent to publication of this Work and authorise the granting of this licence.

For peer review only

Title: Patterns and Determinants of Incident Cataract Surgery in China from 2011 to 2015 Using a Nationally-representative Longitudinal Database

Catherine Jan,[1,2,3] Jin Xin,[4,5] Yanhui Dong,[6] Thomas Butt,[7] Robert T. Chang,[8] Lisa Keay,[9] Ming He,[2,3] David S. Friedman[10,11] and Nathan Congdon[12, 13, 14]

1. Lost Child's Vision Project, Sydney, Australia
2. Centre for Eye Research Australia, Ophthalmology, Department of Surgery, University of Melbourne, Melbourne, Victoria, Australia.
3. Department of Ophthalmology and Surgery, Faculty of Medicine, Dentistry & Health Sciences, University of Melbourne, Melbourne, Australia
4. Institute of Population Research, Peking University
5. Peking University Library, Peking University, Beijing, China
6. Institute of Child and Adolescent Health, School of Public Health, Peking University
7. UCL Institute of Ophthalmology, University College London, London, UK
8. Ophthalmology, Byers Eye Institution at Stanford, Stanford, California, United States
9. School of Optometry and Vision Science, University of New South Wales, Sydney, Australia
10. Department of Ophthalmology, Harvard Medical School, Boston, Massachusetts, USA
11. Massachusetts Eye and Ear Infirmary
12. State Key Laboratory of Ophthalmology, Zhongshan Ophthalmic Center, Sun Yat-sen University, Guangzhou, China
13. ORBIS International, New York, USA
14. TREE Centre, Centre for Public Health, Queen's University Belfast, Belfast, Northern Ireland

For peer review only - http://bmjopen.bmj.com/site/about/guidelines.xhtml

Corresponding author: Catherine Jan, Department of Ophthalmology and Surgery, Faculty of Medicine, Dentistry & Health Sciences, The University of Melbourne, Swanston St PARKVILLE VIC Australia 3052, jlingxue@gmail.com

Key words: Cataract surgery, epidemiology, healthcare access, equity,

For peer review only

For peer review only - http://bmjopen.bmj.com/site/about/guidelines.xhtml

Abstract

Objectives

To investigate incident cataract surgery, and to investigate determinants of cataract surgery uptake in Chinese adults.

Design

This nationally-representative longitudinal study recorded self-reported incident cataract surgery, and measured biological, clinical, and socioeconomical characteristics at baseline and endline.

Setting

In the first stage, 150 county-level units were randomly chosen with a probability-proportional-to-size sampling technique from a sampling frame containing all county-level units. The sample was stratified by region and within region by urban district or rural county and per capita gross domestic product (GDP). The final sample of 150 counties fell within 28 provinces of China.

Participants

Urban and rural Chinese persons aged 45 years and older

Primary and secondary outcome measures

Incident cataract surgery (primary outcome) and the factors associated with incident cataract surgery (secondary outcome).

Results

Among 16,663 people enrolled in 2011, 13,705 (82.2%) attended follow-up in 2015. Among these, 167 (1.22%) reported incident cataract surgery. Those receiving surgery were significantly older (66.2+/-8.79 vs 58.3+/-9.18, p=<0.001), and more likely to report: illiteracy (44.9% vs 27.1%, p<0.001), poor baseline distance vision (49.7% vs 20.0%, p=<0.001), poor baseline near vision (37.1% vs 21.8%, p=<0.001), baseline visual impairment (15.6% vs 5.5%, p=<0.001), diabetes (12.0% vs 7.42% ,p=<0.05), and higher baseline depression scores (9.7 vs 8.4 on a scale of 0-30, p=<0.05).
In linear regression models, older age, worse distance vision, hypertension or diabetes, illiteracy, and lower depression score were significantly associated with undergoing surgery. Results were similar in models including only persons aged ≥60 years, except that urban residence was also associated with

For peer review only - http://bmjopen.bmj.com/site/about/guidelines.xhtml

surgery. When only those aged ≥60 years with poor vision were included, results were again the same, except that higher household expenditure was also associated with surgery.

Conclusions

In China, cataract surgical rates remain low, underserved groups such as rural dwellers are less likely to receive cataract surgery.

Strengths and limitations of this study

- The strengths of this study include a large nationally representative sample from most provinces of China, selected using standardized protocols.
- Study personnel were trained in standard fashion to ensure all procedures were carried out as described.
- Our findings provide a basis for comparison with other low- and middle-income countries and especially those undergoing rapid economic transitions.
- Limitations include that the study did not measure objective visual acuity due to time and cost. Another limitation is that our main outcome, having undergone cataract surgery, depended on patient report for similar reasons, though evidence has suggested that self-reported cataract surgery is accurate and reliable.

Synopsis

In China, 4-year incident cataract surgery remains as low as 1.22% and there is room for improvement.

For peer review only

For peer review only - http://bmjopen.bmj.com/site/about/guidelines.xhtml

Introduction

Blindness is among the most feared health conditions, ranking only after cancer and AIDS, and vision is the most valued of all the senses.[1] Age-related cataract is the world's and China's leading cause of blindness.[2,3] Surgery is currently the only available treatment for cataract, and has been rated among the most cost-effective procedures in medicine by the World Health Organization (WHO).[4] Cataract surgery improves quality of life, increases activities of daily living, and results in higher household economic productivity.[5,6] With a rapidly ageing population in China,[7] a significant increase in the burden of cataract is expected, highlighting the importance of efforts to address this burden.

Previous population-based, cross-sectional studies have reported the rates of prior cataract surgery in different regions of China,[8-12] with population 5-year incidence ranging from 2.9% in the Beijing Eye Study to 4.4% in Liwan, southern China. Rural cataract surgical coverage (CSC, proportion of individuals with vision-impairing cataract who have received surgery) for those with vision of <6/60 rose from 35% (range 24-62%) in 2006[8] to 63% (range 43-84%) in 2014, though coverage varied widely between provinces and was generally lower in poorer, western areas.[13] National prevalence and incidence data across China are vital for effective policy formulation and resource allocation. However, there have been no published studies of cataract surgery incidence using nationally-representative samples in China [14, 15] and most reports are from high-income countries.[16-18]

The multiple barriers to accessing cataract surgery in China include cost (direct out-of-pocket surgical expense as well as transportation and accommodation[19,20]), lack of knowledge about cataract and cataract surgery,[20,21] and concerns about the quality of local surgeons.[22] Prior work in this area has several shortcomings. Firstly, no previous study has used a nationally representative sample which is needed because in a large country like China, social economic status (SES) and other determinants of cataract surgery uptake may vary widely by region. Secondly, many existing studies[19,20,22] have used data from prior to 2009, when China announced systematic

For peer review only - http://bmjopen.bmj.com/site/about/guidelines.xhtml

health reforms. Access to cataract surgery in urban and rural areas has improved substantially due to universal coverage under the Urban Resident Medical Insurance (URMI) and New Cooperative Medical System (NCMS) schemes.[23] Few recent publications using national data on cataract surgery have been published.[23] Finally, while evidence from low-and middle-income countries suggests that health decisions are usually made at the household level, most existing studies in China have included only patient-level data, mostly concerning clinical rather than socio-economic variables.[19]

To address these limitations, the present paper employed a national longitudinal survey conducted in 30 out of 31 mainland Chinese provinces (except Tibet, though the study did include one Tibetan county), representative of the adult population 45 years and older, in order to understand patterns of cataract surgery incidence across China between 2011 and 2015. The aim was to investigate incident cataract surgery, and to investigate the clinical, biological, and socio-economic determinants of and barriers to cataract surgery in adults aged 45 years and above in urban and rural China between 2011 and 2015.

Methods:

Study population

The China Health and Retirement Longitudinal Study (CHARLS) is a nationally-representative longitudinal survey among Chinese persons aged 45 years and older, that includes assessments of biological, social and economic conditions. We used data from the baseline study conducted between July and September 2011 ("Wave 1") and a follow up conducted (both surveys conducted in person) between July and September 2015 ("Wave 3"). Main respondents and spouses in the baseline survey are followed throughout the life of CHARLS, or until they die.[24] Detailed information on the methodology of CHARLS has been described elsewhere.[24] In brief, 17,708 people aged 45 years and older participated in the baseline study in 2011. Among them, 16,663 (94.1%) were eligible for data collection, 107 (0.604%) did not provide information on cataract surgery, 457 (2.58%) were aged

For peer review only - http://bmjopen.bmj.com/site/about/guidelines.xhtml

below 45 years, and 481 (2.72%) had already undergone cataract surgery before the baseline and were excluded from our sample. Among those eligible at baseline who provided information on cataract surgery in 2011 (n=16991, 96.0% of the total), 13705 (82.2%) attended the four-year follow-up in 2015. The protocols of the follow-up for the variables used in this paper followed those of the baseline study.

Samples were chosen through multistage probability sampling. In the first stage, 150 county-level units were randomly chosen with a probability-proportional-to-size (PPS) sampling technique from a sampling frame containing all county-level units with the exception of the Tibet Autonomous Region. The sample was stratified by region and within region by urban district or rural county and per capita statistics on gross domestic product (GDP). The final sample of 150 counties fell within 28 provinces. Our sample used the lowest level of government organization, consisting of administrative villages (*cun*) in rural areas and neighborhoods (*shequ* or *juweihui*) in urban areas, as primary sampling units (PSUs). We selected 3 PSUs within each county-level unit, using PPS sampling (more details are reported elsewhere).[24]

Variables

Cataract surgery incidence refers to the proportion of the cohort population aged 45 years and older who underwent cataract surgery in at least one eye between 2011 and 2015, among those who had not undergone cataract surgery in either eye at baseline. This was determined by participants' response to the question "Have you had cataract surgery before?", comparing answers between the previous and present round.

Age and gender were self-reported variables taken from the baseline 2011 survey. Distance and near vision were self-reported at baseline, using a scale of 1 to 5, with 1 being excellent and 4 and 5 being fair and poor respectively. Binary visual impairment was also self-reported.

For peer review only - http://bmjopen.bmj.com/site/about/guidelines.xhtml

Rural or urban place of residence was obtained from self-reported *Hukou*. *Hukou* is a Chinese internal passport system that is designed to regulate the influx of rural residents into urban areas, and migrants with rural *hukou* do not have equal access to health care compared to local urban residents. Hukou also limits the type of public health insurance the person has access to.

Three comorbidities linked with cataract and/or uptake of cataract surgery were extracted from CHARLS. Hypertension was defined as either self-report of a physician's diagnosis, or presence of objective biomarkers (systolic pressure >= 140 and/or diastolic pressure >= 90 mmHg) during direct examination. Diabetes was similarly defined by self-report of prior diagnosis by a doctor or the presence of a biomarker (HbA1c value ≥6.5%) on examination. Depression was measured using the Center for Epidemiologic Studies Depression Scale (CES-D-10)[25] with total score ranging from 0 (minimal symptoms) to 30 (most symptomatic).

Data were obtained from the National School of Development (China Centre for Economic Research) of Peking University. Secondary analysis of data from CHARLS did not require ethical approval. GDP data were extracted from the China Statistical Yearbook, and per capita household expenditure were derived from surveys of household expenditure divided by the number of household members. GDP in Chinese Yuan was converted to the US dollar using the exchange rate on 1st August, 2011, released by the center for foreign trade, Bank of China.

All data collected in the CHARLS are maintained at the National School of Development of Peking University, Beijing, China. The datasets are available from https://charls.charlsdata.com/pages/data/111/zh-cn.html.[26] The CHARLS was approved by the Ethical Review Committee of Peking University, and all participants gave written informed consent at the time of participation. This study used public data and therefore does not require additional ethics approval. The original CHARLS study from which the data were collected was carried out in Beijing and has obtained ethics approval from the Ethical Review Committee of Beijing University the ethical

For peer review only - http://bmjopen.bmj.com/site/about/guidelines.xhtml

approval number is IRB00001052-11015.

Patient and Public Involvement (PPI)

As this is an analysis of publicly available dataset, neither ethics nor PPI are applicable.

Statistical analysis

All statistical analyses were performed using Stata 13.0 (StataCorp., College Station, TX). The t-test was used to compare continuous variables, while Pearson X^2 or Fisher's exact tests were used for the comparison of categorical data. As a test to assess accuracy of household expenditure data from the sample as an index of income in the province, a scatterplot graph was made comparing household expenditure to provincial GFDP data. The 4-year incidence of cataract surgery stratified by age and gender were calculated. Age was defined in our analysis as the age at baseline. Univariate and multivariate logistic regression models were used to test for association with incident cataract surgery over the 4-year follow-up period. We ran three sets of regression analysis, one including the whole sample of eligible participants (i.e., all eligible people who were aged 45 years and above), one including those eligible participants aged 60 years and above (as visually-significant cataract is far more prevalent in this age range), and the third including those eligible participants aged 60 years and above who also reported fair or poor vision (the subset of participants at greatest risk for undergoing cataract surgery) . The variables in univariate models that had p value of less than 0.05 were deemed significant and were kept in the multivariate models. The incidence of cataract surgery in each province was affected by the age and gender structure of the population aged 45 years and over. Therefore, the provincial incidence of cataract surgery was adjusted for age and gender structure of the population. We applied sample weights to the cataract surgery incidence between 2011 and 2015. These weights were constructed from weights for the structure of age and gender from the baseline wave of the CHARLS study. A p value of <0.05 was defined as statistically significant.

For peer review only - http://bmjopen.bmj.com/site/about/guidelines.xhtml

Results:

Among the 16,663 eligible people aged 45 years and above (Table 1), 13,705 (82.2%) attended the year four follow-up. Among them, 13,538 (98.8%) did not report receiving surgery between 2011 and 2015, while 167 (1.22%) did. Those who received surgery were significantly older (66.17+/-8.79 vs 58.29+/-9.18, p=<0.001), and more likely to be illiterate (45% vs 27%, p<0.001), have self-reported poor distance vision at baseline (49.7% vs 20.0%, p=< 0.001), self-reported poor near vision at baseline (37.1% vs 21.8%, p=< 0.001), self-reported visual impairment at baseline (15.6% vs 5.5%, p=< 0.001), diabetes (12.0% vs 7.4% ,p=< 0.05), and higher depression scores at baseline (9.70 vs 8.37 on a scale of 0-30, p=< 0.05)

Table 1: Baseline characteristics of participants aged 45 and above who participated both the baseline and follow-up surveys.

**Baseline characteristic N=13705， >=45， drop missing cases**	**Cataract surgery N=167**	**No cataract surgery N=13538**
Age (years), mean (Std. Dev.)	66.17(8.79)	58.29(9.18)
Distance Vision status, n (%)		
Poor or fair	125 (75.9)	7782 (57)
Excellent, very good and good	32 (19.2)	4904 (36)
Hypertension, n (%)		
Yes	67 (40),	5238 (39)
No	76 (46)	6532 (48)
Diabetes, n (%)		
Yes	20 (12)	1004 (7.4)
No	102 (61)	8701 (64)

For peer review only - http://bmjopen.bmj.com/site/about/guidelines.xhtml

Male	71(42)	6556(48)
Female gender, n (%)	96(58)	6982(52)
Literacy, n (%)		
Literate	92(55)	9865(73)
Illiterate	75(45)	3673(27)
mean depression score, mean (SD)	9.70(6.53)	8.37(6.30)
Hukou, n (%)		
Rural	127（76.0）	10961（80）
Non-rural	39(23)	2484(18)
Log household per capita expenditure (USD), mean (SD)	4.57 (.94)	4.68(.88)
Health insurance, n (%)		
Yes	158 (95)	12664(94)
No	8 (5)	797(6)
Geographical location, n (%)		
Eastern region	52(31)	4704(35)
Central region	60(36)	4428(33)
Western region	55(33)	3306(34)
marital status		
married	131(78)	11066(82)
Not married	36 (22)	2472 (18)

Table 2 lists the 28 provinces for which data are available in the current study in order of per capita GDP by region and by province within region. There was a non-significant trend towards higher mean weekly household per

For peer review only - http://bmjopen.bmj.com/site/about/guidelines.xhtml

capita expenditure in Eastern China (US$34.3) versus Central (US$20.5) and Western China (US$18.1). Mean provincial per capita weekly household expenditures as reported by study participants were highly correlated ($R2=0.650$, $P=0.0018$, regression correlation) with available data for per capita GDP by province (Figure 1).

Table 2: Characteristics of 28 Chinese provinces with regard to per capita gross domestic product (GDP), population and other characteristics, in 2011 (Arranged by region and province in order of reported per capita GDP)

Province name	Reported per capita GDP （USD, 2011）	Reported total population (thousands) (2011)	Reported population Density (persons/ square kilometre) (2011)	Number of participants in the current study （N=16663）	Mean weekly household per capita expenditure of study participants (n=16,663), 2011 (USD)	Mean household per capita expenditure, 2011, by geographic region (USD)
Eastern China						
Tianjin	13232.0	13,550	2636	149	44.9	34.3
Beijing	12680.0	20,190	1428	85	73.8	
Shanghai	12820.1	23,470	3702	58	58.6	
Jiangsu	9672.5	78,990	2013	834	38.8	
Zhejiang	9200.3	54,630	1741	659	48.0	
Guangdong	7889.4	105,050	2637	913	31.9	
Liaoning	7882.1	43,830	1712	510	24.3	
Fujian	7356.8	37,200	2306	483	36.5	
Shandong	7350.3	96,370	1389	1374	24.4	
Jilin	5972.1	27,490	2371	415	32.6	
Hebei	5274.8	72,410	2362	712	24.1	
Heilongjiang	5096.2	38,340	5146	312	39.5	

For peer review only - http://bmjopen.bmj.com/site/about/guidelines.xhtml

TOTAL	8702.2	50,960	2454	6504	39.8	
Central China						
Hubei	5310.2	57,580	1969	518	27.7	20.5
Shanxi	4869.2	35,930	2977	511	22.3	
Hunan	4639.8	65,960	2908	773	28.3	
Henan	4450.5	93,880	5124	1313	24.7	
Jiangxi	4060.6	44,880	4527	815	29.7	
Anhui	3984.4	59,680	2265	816	23.4	
TOTAL	4552.5	59,652	3295	4746	26.0	
Western China						
Inner Mongolia	9002.3	24,820	764	819	44.3	18.1
Chongqing	5357.2	29,190	1830	219	29.5	
Shaanxi	5196.4	37,430	5821	532	45.3	
Xinjiang	4672.0	22,090	4563	96	26.2	
Qinghai	4584.2	5680	2487	144	25.1	
Sichuan	4058.0	80,500	2782	1433	25.7	
Guangxi	3932.7	46450	1569	582	22.2	
Gansu	3042.7	25640	3824	402	24.8	
Yunnan	2991.5	46310	3811	997	21.7	
Guizhou	2548.6	34690	3502	189	23.9	
TOTAL	4538.6	35,280	3095	5413	28.9	

Among 7052 (42.3%) participants in the current study aged 60 and above, 126 indicated they had undergone cataract surgery during 2011 to 2015. Among 4522 (27.1%) participants aged 60 years and above and reporting

For peer review only

"fair" or "poor" vision at the time of the most recent survey four years previously, data on cataract surgery were missing for 38 persons (0.840%), and a total of 98 (2.19% or 98/4484) indicated they had undergone cataract surgery, the 4-year incidence rate ranging from 2.43% (31/1401) in Central China, to 2.27% (35/1545) in the East to 2.08% (32/1538) in the West. (Table 3)

Table 3: Characteristics of persons aged 60 and over with self-reported vision of fair or poor in 2011 (N=4522) by region. Regions are listed in order of per capita GDP in 2011.

Economic region	Mean age (SD)	Women, n (%)	Literate, n (%)*	Mean depression score (95%CI) (SD)***	Rural (versus urban) residence, n (%)*	Mean household per capita expenditure, USD (SD)	Has only public health insurance, n (%)***	4-year cataract surgery incidence****, (%)
Eastern region	68.7 (0.181)	814 (52.7)	978 (63.3%)	9.18 （8.86-9.50） (0.164)	1,151 (74.6%)	24.7 (1.05)	1399 （90.6）	35/1,545 (2.27%)
Central region	67.8 (0.177)	705 (50.3%)	840 (60.0%)	10.1 （9.78-10.49） (0.179)	1,130 (80.7%)	19.7 (0.728)	1292 （92.2）	31/1,401 (2.43%)
Western region	68.4 (0.170)	791 (51.4%)	909 (59.1%)	10.9 （10.57-11.24） （0.17）	1,228 (79.8%)	21.2 (0.854)	1397 （90.8）	32/1,538 (2.08%)
TOTAL	68.34 (0.101)	2,327 (51.46%)	2,727/4484	10.07	3509/4482	21.8	4088/4422	98/4484 (2.19%)

For peer review only - http://bmjopen.bmj.com/site/about/guidelines.xhtml

			(60.82%)	(9.88-10.23) (.099)	(78.29%)	(0.5)	(92.45%)	

* P< 0.05, ** P < 0.01, *** P < 0.001, Chi square test for trend from highest to lowest cataract surgery incidence

****Data on cataract surgery was missing for 38 persons (0.840%)

For peer review only

For peer review only - http://bmjopen.bmj.com/site/about/guidelines.xhtml

In linear regression models of potential predictors of regional incidence of cataract surgery among persons aged 45 years and above at baseline, older age, worse vision, having hypertension or diabetes, being illiterate, and higher depression score were all significantly associated with increased surgery rates in the univariate model, and all these factors remained significant in multivariate model (Table 4). Results were similar in the multivariate regression model including only persons aged 60 years and above, except that urban hukou was also associated with higher incidence of surgery (Table 5). When only those aged 60 years and above with self-reported fair or poor vision were included, results in the multivariate model were again the same, except that higher household expenditure was also associated with higher incidence of surgery (Table 6).

Table 4: Linear regression model of potential predictors of the incidence of cataract surgery among all persons aged > =45 years (n= 16663, among whom 167 [1.00%] reported having surgery) across whole of China between 2011 and 2015 **(Bold type indicates values significant at P<0.05)**

Baseline Characteristic	Univariate model			Multivariate model		
	Beta coefficient	95% Confidence Interval	P-value	Beta coefficient	95% Confidence Interval	P-value
Age	**.094**	**-.0013 -.0009**	**<0.001**	**.080**	**.00098 .00106**	**<0.001**
Poor or Fair self-reported vision	**0.41**	**.005 .013**	**<0.001**	**.022**	**00485 .00625**	**<0.001**
Hypertension present	**.005**	**.001 .002**	**<0.001**	**-.018**	**-.0048 -.0035**	**<0.001**

For peer review only - http://bmjopen.bmj.com/site/about/guidelines.xhtml

(adjusted for age)						
Diabetes present (adjusted for age)	**.023**	**.008 .010**	**<0.001**	**.019**	**.0063 .0085**	**<0.001**
Female sex	.013	-.001 .007	0.129			
Literate	**-.0438**	**-.015 .007**	**<0.001**	**-.021**	**-.00614 -.00468**	**<0.001**
Mean depression score (Adjusted for age)	**.023**	**.0004 .0005**	**<0.001**	**.012**	**.00017 .00028**	**<0.001**
Rural (vs non-rural) hukou	-.014	-.0008 .0085	0.106			
Log household per capita expenditure	-.013	-.0038 .0006	0.146			
Reported having health insurance	.005	-.0054 .0102	0.550			

Table 5: Linear regression model of potential predictors of the regional incidence of cataract surgery among all persons aged > =60 years (n= 7052, among whom 126 [1.79%] reported having surgery) **(Bold type indicates values significant at P<0.05)**

Baseline Characteristic	Univariate model			Multivariate model*		
	Beta coefficient	95% Confidence Interval	P-value	Beta coefficient	95% Confidence Interval	P-value
Age	**0.060**	**.0007， .020**	**<0.001**	**.036**	**.00077 .00096**	**<0.001**
Poor or Fair self-reported vision	**.052**	**-.025 -.008**	**<0.001**	**.039**	**.01167 .01431**	**<0.001**
Hypertension present (adjusted for age)	**-.011**	**-.0043 -.0023**	**<0.001**	**-.023**	**-.00804 - .00567**	**<0.001**
Diabetes present (adjusted for age)	**.029**	**.0117 .0151**	**<0.001**	**.024**	**.00949 .01318**	**<0.001**
Female sex	.024	-.00005 .01499	0.068			
Literate	**-.032**	**-.0178 .0017**	**0.018**	**-.027**	**-.00957 - .00701**	**<0.001**
Mean depression score (Adjusted for age)	**.020**	**.000383 -.000536**	**<0.001**	**.023**	**.00044 .00062**	**<0.001**

For peer review only - http://bmjopen.bmj.com/site/about/guidelines.xhtml

Rural (vs non-rural) hukou	**-.030**	**-.0204 -.0015**	**0.024**	**-.043**	**-.01801 - .01489**	**<0.001**
Log household per capita expenditure	.011	-.00277 .00642	0.435			
Reported having health insurance	.001	-.016 .017	0.926			

For peer review only

1 Table 6: Linear regression model of potential predictors of the regional incidence of cataract surgery among all
2 persons aged > =60 years and those who reported fair or poor vision (n= 4522) **(Bold type indicates values**
3 **significant at P<0.05)**

Baseline Characteristic	Univariate model			Multivariate model*		
	Beta coefficient	95% Confidence Interval	P-value	Beta coefficient	95% Confidence Interval	P-value
Age	**.064**	**.0008227 .002492**	**<0.001**	**.035**	**.00076 .00101**	**<0.001**
Hypertension present (adjusted for age)	**-.014**	**-.00596 -.0032489**	**<0.001**	**-.039**	**-.01425 -.01100**	**<0.001**
Diabetes present (adjusted for age)	**.041**	**.0184 .0229**	**<0.001**	**.041**	**.01744 .02250**	**<0.001**
Female sex	.024	-.00278 .01839	0.148			
Literate	-.027	-.01995 .00171	0.099			
Mean depression score (Adjusted for age)	**.006**	**.0000485 .0002474**	**0.004**	**.027**	**.00053 .00078**	**0.004**
Rural (vs non-rural) hukou	**-.041**	**-.030558 -.003757**	**0.012**	**- .031**	**-.01515 -.01066**	**<0.001**

For peer review only - http://bmjopen.bmj.com/site/about/guidelines.xhtml

Log household per capita expenditure	**.036**	**.0001394 .012749**	**0.045**	**.012**	**.00130 .0032**	**<0.001**
Reported having health insurance	-.001	-.022986 .021785	0.958			

Discussion:

Between 2011 and 2015, the incidence of self-reported cataract surgery in the current study was relatively low (2.19%) among people aged 60 and above compared to earlier reports, which ranged from 2.9% (5-year incidence) in the Beijing Eye Study[10] to 4.4% (5-year incidence) in Liwan, southern China.[12] More data are needed to help policy makers address the barriers to uptake of cataract surgery in China.[19-22] Previous studies have examined institutional predictors of higher surgical capacity at the institutional level (principally screening outreach activities). This is first large population study of determinants of surgery uptake in China, providing complementary information.

Our finding of lower surgical rates in more rural provinces is in agreement with results from prior population studies[8,13] which have also reported especially low rates of surgical uptake in rural settings. This finding indicates that this lower rate persists despite the rapid expansion of the NCMS rural health insurance program.[27,28]

For peer review only - http://bmjopen.bmj.com/site/about/guidelines.xhtml

With respect to individual-level variables, it was unsurprising that worse self-reported vision, as captured by three different variables, was significantly associated with greater uptake of surgery. As expected, older age was associated with elevated uptake of surgery.

Diabetes is well-established in population studies to be associated with higher risk of lens opacity, and so our observed higher prevalence of diabetes among those accepting surgery is not surprising. Cataract has also been linked with higher rates of mental health problems, including depression,[29] which we believe explains the observed higher rates of depression among those undergoing incident cataract surgery. An unexplained individual-level finding was a higher prevalence of self-reported illiteracy among those accepting incident cataract surgery. Poor education and lack of knowledge about cataract have generally been associated with low surgical uptake in previous population studies.[22-23] One possible speculation could be that illiterate people were more likely to do what the doctor recommended. Those with better education may have been more wary of using local services but could not afford to go to more distant locations for care. Alternatively, those with lower literacy may have other unmeasured factors that predispose to higher rates of cataract such as greater exposure to UV light.

Higher economic status (represented by higher weekly household per capita expenditure, which correlated strongly with per capita GDP, Fig 1) was associated with receiving cataract surgery. This result is expected, however, there have been several efforts in recent years that help provide surgery to those economically more disadvantaged. One is the roll-out of projects providing high-volume free surgery, such as the Million People Cataract Surgery Plan,[30] during the period covered in the current study. This has been supplemented by non-governmental organization (NGO)-supported cataract surgical programs by entities such as Lifeline Express,[31] and by the high penetration of the NCMS rural health insurance system, now covering some 98.9% of rural-dwellers[27] and substantially reducing out-of-pocket expenses associated with cataract surgery. We observed that the percentages of people covered by public health insurance did not vary significantly between poorer and richer

For peer review only - http://bmjopen.bmj.com/site/about/guidelines.xhtml

regions. These programs taken together may have improved access in poor areas that reduced inequality in cataract surgery access associated with income, but in the end, those with greater income were more likely to have cataract surgery.

The strengths of this study include a large nationally representative sample from most provinces of China, selected using standardized protocols. Study personnel were trained in standard fashion to ensure all procedures were carried out as described. Our findings provide a basis for comparison with other low- and middle-income countries and especially those undergoing rapid economic transitions. Limitations must be acknowledged as well. In the first place, the study did not measure objective visual acuity due to time and cost. Our main outcome, having undergone cataract surgery, depended on patient report for similar reasons, though evidence has suggested that self-reported cataract surgery is accurate and reliable.[32] Finally, the results may be influenced by patients (17.8%) who did not come for the follow-up examination.

Conclusion:

Our study provides unique data on cataract surgical incidence in China. Our results suggest that surgical rates remain low. Rural and metropolitan both have access to cataract surgery, but underserved groups such as rural dwellers are less likely to receive cataract surgery.

Acknowledgement:

The authors would like to thank Professor Zhao Yaohui for her valuable comments and suggestions on this manuscript. We would also like to thank the CHARLS team for the data that enabled these analyses.

Author Contribution

For peer review only

Design: Jan and Congdon; Draft manuscript: Jan; Data analysis: Jan and Jin; Interpret data: Jan, Jin, Congdon, Friedman, Butt, Keay, He; Revise manuscript: Jin, Congdon, Butt, Dong, Friedman, Chang, Keay, He.

Competing interests: The authors have no competing interest.

Funding: N/A

Data sharing statement: All data collected in the CHARLS are maintained at the National School of Development of Peking University, Beijing, China. The datasets are available from http://charls.pku.edu.cn/pages/data/111/zh-cn. html.

Ethics approval statement: The CHARLS was approved by the Ethical Review Committee of Peking University, and all participants gave written informed consent at the time of participation. This study used public data and therefore does not require additional ethics approval. The original CHARLS study from which the data were collected was carried out in Beijing and has obtained ethics approval from the Ethical Review Committee of Beijing University the ethical approval number is IRB00001052-11015.

For peer review only - http://bmjopen.bmj.com/site/about/guidelines.xhtml

References

1. Enoch J, McDonald L, Jones L, Jones PR, Crabb DP. Evaluating whether sight is the most valued sense. *JAMA Ophthalmol* 2019; **137**(11): 1317-20.

2. Bourne RR, Stevens GA, White RA, et al. Causes of vision loss worldwide, 1990-2010: a systematic analysis. *Lancet Glob Health* 2013; **1**(6): e339-49.

3. Liang YB, Friedman DS, Wong TY, et al. Prevalence and causes of low vision and blindness in a rural Chinese adult population: the Handan Eye Study. *Ophthalmology* 2008; **115**(11): 1965-72.

4. Horton S, Gelband H, Jamison D, Levin C, Nugent R, Watkins D. Ranking 93 health interventions for low- and middle-income countries by cost-effectiveness. *PLoS One* 2017; **12**(8).

5. Lee BS, Munoz BE, West SK, Gower EW. Functional improvement after one-and two-eye cataract surgery in the Salisbury Eye Evaluation. *Ophthalmology* 2013; **120**(5): 949-55.

6. Wang JJ, Mitchell P, Smith W. Vision and low self-rated health: the Blue Mountains Eye Study. *Invest Ophthalmol Vis Sci*. 2000; **41**(1): 49-54.

7. Lopreite M, Zhu Z. The effects of ageing population on health expenditure and economic growth in China: A Bayesian-VAR approach. Social science & medicine. 2020 Nov 1;265:113513.

8. Zhao J, Ellwein LB, Cui H, et al. Prevalence and outcomes of cataract surgery in rural China: the China nine-province survey. *Ophthalmology* 2010; **117**(11): 2120-8.

9. Lavanya R, Wong TY, Aung T, et al. Prevalence of cataract surgery and post-surgical visual outcomes in an urban Asian population: the Singapore Malay Eye Study. *Br J Ophthalmol*. 2009; **93**(3): 299-304.

10. Liu B, Xu L, Wang YX, Jonas JB. Prevalence of cataract surgery and postoperative visual outcome in Greater Beijing: the Beijing Eye Study. *Ophthalmology* 2009; **116**(7): 1322-31.

11. Li EY, Liu Y, Zhan X, et al. Prevalence of blindness and outcomes of cataract surgery in Hainan Province in South China. *Ophthalmology* 2013; **120**(11): 2176-83.

For peer review only - http://bmjopen.bmj.com/site/about/guidelines.xhtml

12. Huang W, Huang G, Wang D, Yin Q, Foster PJ, He M. Outcomes of cataract surgery in urban southern China: the Liwan Eye Study. *Invest Ophthalmol Vis Sci*. 2011; **52**(1): 16-20.

13. Zhao J, Xu X, Ellwein LB, et al. Cataract Surgical Coverage and Visual Acuity Outcomes in Rural China in 2014 and Comparisons With the 2006 China Nine-Province Survey. *Am J Ophthalmol* 2018; **193**: 62-70.

14. Zhang JS, Xu L, Wang YX, You QS, Da Wang J, Jonas JB. Five-year incidence of age-related cataract and cataract surgery in the adult population of greater Beijing: the Beijing Eye Study. *Ophthalmology* 2011; **118**(4): 711-8.

15. Huang W, Zheng Y, Wang L, et al. Five-year incidence and postoperative visual outcome of cataract surgery in urban southern China: the Liwan Eye Study *Invest Ophthalmol Vis Sci*. 2012; **53**(13): 7936-42.

16. Kanthan GL, Wang JJ, Rochtchina E, et al. Ten-year incidence of age-related cataract and cataract surgery in an older Australian population: the Blue Mountains Eye Study. *Ophthalmology* 2008; **115**(5): 808-14.

17. Klein BEK, Klein R, Lee KE. Incidence of age-related cataract over a 10-year interval: the Beaver Dam Eye Study. *Ophthalmology* 2002; **109**(11): 2052-7.

18. Koo E, Chang JR, Agrón E, et al. Ten-year incidence rates of age-related cataract in the Age-Related Eye Disease Study (AREDS): AREDS report no. 33. *Ophthalmic Epidemiol* 2013; **20**(2): 71-81.

19. Li Z, Song Z, Wu S, et al. Outcomes and barriers to uptake of cataract surgery in rural northern China: the Heilongjiang Eye Study. *Ophthalmic Epidemiol* 2014; **21**(3): 161-8.

20. Zhang M, Wu J, Li L, et al. Impact of cataract screening outreach in rural China. *Invest Ophthalmol Vis Sci* 2010; **51**(1): 110-4.

21. Yin Q, Hu A, Liang Y, et al. A two-site, population-based study of barriers to cataract surgery in rural China. *Invest Ophthalmol Vis Sci*. 2009; **50**(3): 1069-75.

22. Zhang M, Wu X, Li L, et al. Understanding barriers to cataract surgery among older persons in rural China through focus groups. *Ophthalmic Epidemiol* 2011; **18**(4): 179-86.

For peer review only - http://bmjopen.bmj.com/site/about/guidelines.xhtml

23. An L, Jan CL, Feng J, Wang Z, Zhan L, Xu X. Inequity in access: cataract surgery throughput of Chinese ophthalmologists from the China national eye care capacity and resource survey. Ophthalmic Epidemiology. 2020 Jan 2;27(1):29-38.

24. Zhao Y, Hu Y, Smith JP, Strauss J, Yang G. Cohort profile: the China health and retirement longitudinal study (CHARLS). *Int J Epidemiol*. 2014; **43**(1): 61-8.

25. Lewinsohn PM, Seeley JR, Roberts RE, Allen NB. Center for Epidemiologic Studies Depression Scale (CES-D) as a screening instrument for depression among community-residing older adults. *Psychol Aging*. 1997 Jun;12(2):277.

26. (Dataset) https://charls.charlsdata.com/pages/data/111/zh-cn.html (Last accessed 3 Feb 2023)

27. Gu D, Yang X, Li X, Liang C, Zhong J, Feng N. Innovating new rural cooperative medical scheme (NCMS) for better patient satisfaction in rural China. Int J Env Res Pub Health. 2018 Sep;15(9):2007.

28. Drinkwater JJ, Davis WA, Davis TM. A systematic review of risk factors for cataract in type 2 diabetes. Diabetes/metabolism research and reviews. 2019 Jan;35(1):e3073.

29. Pellegrini M, Bernabei F, Schiavi C, Giannaccare G. Impact of cataract surgery on depression and cognitive function: Systematic review and meta-analysis. Clin Exper Ophthalmol. 2020 Mar 27.

30. Yan X, Guan C, Mueller A, Iezzi B, He M, Liang H, Meltzer M, Congdon NG. Outcomes and predicted impact on vision restoration of the China Million Cataract Surgeries Program. *Ophthalmic Epidemiol.* 2013;20:294-300.

31. Lifeline Express home page. http://www.lifeline-express.com/. Last visited 4 December 2020.

32. Bowie H, Congdon N, Lai H, West SK. Validity of a personal and family history of cataract and cataract surgery in genetic studies. *Invest Ophthalmol Clin Sci* 2003;44:2905-8.

For peer review only - http://bmjopen.bmj.com/site/about/guidelines.xhtml

Figure 1: Scatterplot and regression line showing the reported per capita gross domestic product of provinces (USD) against the mean household per capita expenditure (USD) of participants in our study from these provinces.

For peer review only

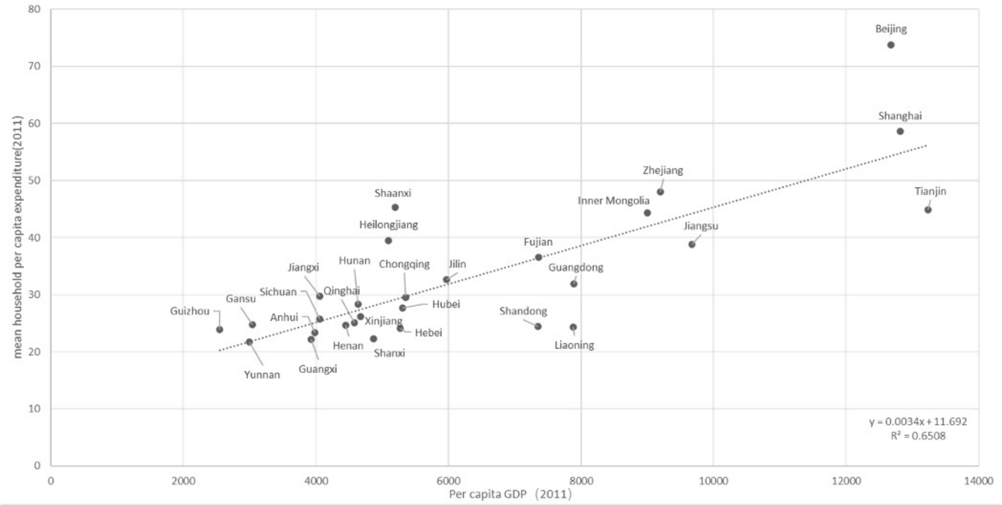

184x93mm (144 x 144 DPI)

STROBE Statement—checklist of items that should be included in reports of observational studies

	Item No.	Recommendation	Page No.	Relevant text from manuscript
**Title and abstract**	1	(*a*) Indicate the study's design with a commonly used term in the title or the abstract	Page 1 lines 1-2	
		(*b*) Provide in the abstract an informative and balanced summary of what was done and what was found	Page 3 lines 1-33, page 4 lines 1-7	
**Introduction**				
Background/rationale	2	Explain the scientific background and rationale for the investigation being reported	Page 5 lines 1-24, page 6 lines 1-6	
Objectives	3	State specific objectives, including any prespecified hypotheses	Page 6 lines 8-13	
**Methods**				
Study design	4	Present key elements of study design early in the paper	Page 6 line 15-24, page 7 lines 1-13	
Setting	5	Describe the setting, locations, and relevant dates, including periods of recruitment, exposure, follow-up, and data collection	Page 7 lines 2-13	
Participants	6	(*a*) *Cohort study*—Give the eligibility criteria, and the sources and methods of selection of participants. Describe methods of follow-up<br>*Case-control study*—Give the eligibility criteria, and the sources and methods of case ascertainment and control selection. Give the rationale for the choice of cases and controls<br>*Cross-sectional study*—Give the eligibility criteria, and the sources and methods of selection of participants	Page 6 lines 22-24, page 7 lines 1-2	
		(*b*) *Cohort study*—For matched studies, give matching criteria and number of exposed and unexposed<br>*Case-control study*—For matched studies, give matching criteria and the number of controls per case	NA	
Variables	7	Clearly define all outcomes, exposures, predictors, potential confounders, and effect modifiers. Give diagnostic criteria, if applicable	Page 7 lines 15-22, page 8 lines 1-17	
Data sources/ measurement	8*	For each variable of interest, give sources of data and details of methods of assessment (measurement). Describe comparability of assessment methods if there is more than one group	Page 7 lines 15-22, page 8 lines 1-17	
Bias	9	Describe any efforts to address potential sources of bias	NA	

For peer review only - http://bmjopen.bmj.com/site/about/guidelines.xhtml

Study size	10	Explain how the study size was arrived at	Page 7 lines 6-13
Quantitative variables	11	Explain how quantitative variables were handled in the analyses. If applicable, describe which groupings were chosen and why	Page 9 lines 7-23
Statistical methods	12	(*a*) Describe all statistical methods, including those used to control for confounding	Page 9 lines 7-23
		(*b*) Describe any methods used to examine subgroups and interactions	NA
		(*c*) Explain how missing data were addressed	NA
		(*d*) *Cohort study*—If applicable, explain how loss to follow-up was addressed<br>*Case-control study*—If applicable, explain how matching of cases and controls was addressed<br>*Cross-sectional study*—If applicable, describe analytical methods taking account of sampling strategy	NA
		(*e*) Describe any sensitivity analyses	NA
**Results**			
Participants	13*	(a) Report numbers of individuals at each stage of study—eg numbers potentially eligible, examined for eligibility, confirmed eligible, included in the study, completing follow-up, and analysed	Page 10 lines 1-8
		(b) Give reasons for non-participation at each stage	NA
		(c) Consider use of a flow diagram	NA
Descriptive data	14*	(a) Give characteristics of study participants (eg demographic, clinical, social) and information on exposures and potential confounders	Page 10 lines 4-8
		(b) Indicate number of participants with missing data for each variable of interest	NA
		(c) *Cohort study*—Summarise follow-up time (eg, average and total amount)	Page 10 lines 2-3
Outcome data	15*	*Cohort study*—Report numbers of outcome events or summary measures over time	Page 10 lines 2-3
		Case-control study—Report numbers in each exposure category, or summary measures of exposure	
		Cross-sectional study—Report numbers of outcome events or summary measures	
Main results	16	(*a*) Give unadjusted estimates and, if applicable, confounder-adjusted estimates and their precision (eg, 95% confidence interval). Make clear which confounders were adjusted for and why they were included	Pages 10-21
		(*b*) Report category boundaries when continuous variables were categorized	Pages 10-21
		(*c*) If relevant, consider translating estimates of relative risk into absolute risk for a meaningful time period	NA

Continued on next page

For peer review only

Other analyses	17	Report other analyses done—eg analyses of subgroups and interactions, and sensitivity analyses	NA
**Discussion**			
Key results	18	Summarise key results with reference to study objectives	Page 21 lines 5-15, page 22 lines 1-24, page 23 lines 1-3
Limitations	19	Discuss limitations of the study, taking into account sources of potential bias or imprecision. Discuss both direction and magnitude of any potential bias	Page 23 lines 8-12
Interpretation	20	Give a cautious overall interpretation of results considering objectives, limitations, multiplicity of analyses, results from similar studies, and other relevant evidence	Page 21 lines 5-15, page 22 lines 1-24, page 23 lines 1-3
Generalisability	21	Discuss the generalisability (external validity) of the study results	NA
**Other information**			
Funding	22	Give the source of funding and the role of the funders for the present study and, if applicable, for the original study on which the present article is based	NA

*Give information separately for cases and controls in case-control studies and, if applicable, for exposed and unexposed groups in cohort and cross-sectional studies.

Note: An Explanation and Elaboration article discusses each checklist item and gives methodological background and published examples of transparent reporting. The STROBE checklist is best used in conjunction with this article (freely available on the Web sites of PLoS Medicine at http://www.plosmedicine.org/, Annals of Internal Medicine at http://www.annals.org/, and Epidemiology at http://www.epidem.com/). Information on the STROBE Initiative is available at www.strobe-statement.org.

For peer review only

Patterns and Determinants of Incident Cataract Surgery in China from 2011 to 2015 Using a Nationally-representative Longitudinal Database

Journal:	*BMJ Open*
Manuscript ID	bmjopen-2022-069702.R2
Article Type:	Original research
Date Submitted by the Author:	21-Mar-2023

Complete List of Authors:	Jan, Catherine; The University of Melbourne Department of Surgery in Ophthalmology, Ophthalmology<br>Xin, Jin; Peking University; Peking University<br>Dong, Yanhui; Peking University<br>Butt, Thomas; University College London<br>Chang, Robert; Stanford University, Ophthalmology<br>Keay, Lisa; University of New South Wales, School of Optometry and Vision Science<br>He, Mingguang; Sun Yat-Sen University Zhongshan Ophthalmic Center; Royal Victorian Eye and Ear Hospital, Centre for Eye Research Australia<br>Friedman, David; Harvard University, Ophthalmology<br>Congdon, Nathan; Queen's University Belfast

**Primary Subject Heading**:	Public health
Secondary Subject Heading:	Ophthalmology
Keywords:	EPIDEMIOLOGY, Cataract and refractive surgery < OPHTHALMOLOGY, Ophthalmology < SURGERY

SCHOLARONE™ Manuscripts

For peer review only - http://bmjopen.bmj.com/site/about/guidelines.xhtml

I, the Submitting Author has the right to grant and does grant on behalf of all authors of the Work (as defined in the below author licence), an exclusive licence and/or a non-exclusive licence for contributions from authors who are: i) UK Crown employees; ii) where BMJ has agreed a CC-BY licence shall apply, and/or iii) in accordance with the terms applicable for US Federal Government officers or employees acting as part of their official duties; on a worldwide, perpetual, irrevocable, royalty-free basis to BMJ Publishing Group Ltd ("BMJ") its licensees and where the relevant Journal is co-owned by BMJ to the co-owners of the Journal, to publish the Work in this journal and any other BMJ products and to exploit all rights, as set out in our licence.

The Submitting Author accepts and understands that any supply made under these terms is made by BMJ to the Submitting Author unless you are acting as an employee on behalf of your employer or a postgraduate student of an affiliated institution which is paying any applicable article publishing charge ("APC") for Open Access articles. Where the Submitting Author wishes to make the Work available on an Open Access basis (and intends to pay the relevant APC), the terms of reuse of such Open Access shall be governed by a Creative Commons licence – details of these licences and which Creative Commons licence will apply to this Work are set out in our licence referred to above.

Other than as permitted in any relevant BMJ Author's Self Archiving Policies, I confirm this Work has not been accepted for publication elsewhere, is not being considered for publication elsewhere and does not duplicate material already published. I confirm all authors consent to publication of this Work and authorise the granting of this licence.

For peer review only

Title: Patterns and Determinants of Incident Cataract Surgery in China from 2011 to 2015 Using a Nationally-representative Longitudinal Database

Catherine Jan,[1,2,3] Jin Xin,[4,5] Yanhui Dong,[6] Thomas Butt,[7] Robert T. Chang,[8] Lisa Keay,[9] Ming He,[2,3] David S. Friedman[10,11] and Nathan Congdon[12, 13, 14]

1. Lost Child's Vision Project, Sydney, Australia
2. Centre for Eye Research Australia, Ophthalmology, Department of Surgery, University of Melbourne, Melbourne, Victoria, Australia.
3. Department of Ophthalmology and Surgery, Faculty of Medicine, Dentistry & Health Sciences, University of Melbourne, Melbourne, Australia
4. Institute of Population Research, Peking University
5. Peking University Library, Peking University, Beijing, China
6. Institute of Child and Adolescent Health, School of Public Health, Peking University
7. UCL Institute of Ophthalmology, University College London, London, UK
8. Ophthalmology, Byers Eye Institution at Stanford, Stanford, California, United States
9. School of Optometry and Vision Science, University of New South Wales, Sydney, Australia
10. Department of Ophthalmology, Harvard Medical School, Boston, Massachusetts, USA
11. Massachusetts Eye and Ear Infirmary
12. State Key Laboratory of Ophthalmology, Zhongshan Ophthalmic Center, Sun Yat-sen University, Guangzhou, China
13. ORBIS International, New York, USA
14. TREE Centre, Centre for Public Health, Queen's University Belfast, Belfast, Northern Ireland

For peer review only - http://bmjopen.bmj.com/site/about/guidelines.xhtml

Corresponding author: Catherine Jan, Department of Ophthalmology and Surgery, Faculty of Medicine, Dentistry & Health Sciences, The University of Melbourne, Swanston St PARKVILLE VIC Australia 3052, jlingxue@gmail.com

Key words: Cataract surgery, epidemiology, healthcare access, equity,

For peer review only

Abstract

Objectives
To investigate incident cataract surgery, and to investigate determinants of cataract surgery uptake in Chinese adults.

Design
This nationally-representative longitudinal study recorded self-reported incident cataract surgery, and measured biological, clinical, and socioeconomical characteristics at baseline and endline.

Setting
In the first stage, 150 county-level units were randomly chosen with a probability-proportional-to-size sampling technique from a sampling frame containing all county-level units. The sample was stratified by region and within region by urban district or rural county and per capita gross domestic product (GDP). The final sample of 150 counties fell within 28 provinces of China.

Participants
Urban and rural Chinese persons aged 45 years and older

Primary and secondary outcome measures
Incident cataract surgery (primary outcome) and the factors associated with incident cataract surgery (secondary outcome).

Results
Among 16,663 people enrolled in 2011, 13,705 (82.2%) attended follow-up in 2015. Among these, 167 (1.22%) reported incident cataract surgery. Those receiving surgery were significantly older (66.2+/-8.79 vs 58.3+/-9.18, p=<0.001), and more likely to report: illiteracy (44.9% vs 27.1%, p<0.001), poor baseline distance vision (49.7% vs 20.0%, p=<0.001), poor baseline near vision (37.1% vs 21.8%, p=<0.001), baseline visual impairment (15.6% vs 5.5%, p=<0.001), diabetes (12.0% vs 7.42% ,p=<0.05), and higher baseline depression scores (9.7 vs 8.4 on a scale of 0-30, p=<0.05).
In linear regression models, older age, worse distance vision, hypertension or diabetes, illiteracy, and lower depression score were significantly associated with undergoing surgery. Results were similar in models including only persons aged ≥60 years, except that urban residence was also associated with

surgery. When only those aged ≥60 years with poor vision were included, results were again the same, except that higher household expenditure was also associated with surgery.

Conclusions

In China, cataract surgical rates remain low, underserved groups such as rural dwellers are less likely to receive cataract surgery.

Strengths and limitations of this study

- The strengths of this study include a large nationally representative sample from most provinces of China, selected using standardized protocols.
- Study personnel were trained in standard fashion to ensure all procedures were carried out as described.
- Our findings provide a basis for comparison with other low- and middle-income countries and especially those undergoing rapid economic transitions.
- Limitations include that the study did not measure objective visual acuity due to time and cost. Another limitation is that our main outcome, having undergone cataract surgery, depended on patient report for similar reasons, though evidence has suggested that self-reported cataract surgery is accurate and reliable.

Synopsis

In China, 4-year incident cataract surgery remains as low as 1.22% and there is room for improvement.

For peer review only

Introduction

Blindness is among the most feared health conditions, ranking only after cancer and AIDS, and vision is the most valued of all the senses.[1] Age-related cataract is the world's and China's leading cause of blindness.[2,3] Surgery is currently the only available treatment for cataract, and has been rated among the most cost-effective procedures in medicine by the World Health Organization (WHO).[4] Cataract surgery improves quality of life, increases activities of daily living, and results in higher household economic productivity.[5,6] With a rapidly ageing population in China,[7] a significant increase in the burden of cataract is expected, highlighting the importance of efforts to address this burden.

Previous population-based, cross-sectional studies have reported the rates of prior cataract surgery in different regions of China,[8-12] with population 5-year incidence ranging from 2.9% in the Beijing Eye Study to 4.4% in Liwan, southern China. Rural cataract surgical coverage (CSC, proportion of individuals with vision-impairing cataract who have received surgery) for those with vision of <6/60 rose from 35% (range 24-62%) in 2006[8] to 63% (range 43-84%) in 2014, though coverage varied widely between provinces and was generally lower in poorer, western areas.[13] National prevalence and incidence data across China are vital for effective policy formulation and resource allocation. However, there have been no published studies of cataract surgery incidence using nationally-representative samples in China [14, 15] and most reports are from high-income countries.[16-18]

The multiple barriers to accessing cataract surgery in China include cost (direct out-of-pocket surgical expense as well as transportation and accommodation[19,20]), lack of knowledge about cataract and cataract surgery,[20,21] and concerns about the quality of local surgeons.[22] Prior work in this area has several shortcomings. Firstly, no previous study has used a nationally representative sample which is needed because in a large country like China, social economic status (SES) and other determinants of cataract surgery uptake may vary widely by region. Secondly, many existing studies[19,20,22] have used data from prior to 2009, when China announced systematic

For peer review only - http://bmjopen.bmj.com/site/about/guidelines.xhtml

health reforms. Access to cataract surgery in urban and rural areas has improved substantially due to universal coverage under the Urban Resident Medical Insurance (URMI) and New Cooperative Medical System (NCMS) schemes.[23] Few recent publications using national data on cataract surgery have been published.[23] Finally, while evidence from low-and middle-income countries suggests that health decisions are usually made at the household level, most existing studies in China have included only patient-level data, mostly concerning clinical rather than socio-economic variables.[19]

To address these limitations, the present paper employed a national longitudinal survey conducted in 30 out of 31 mainland Chinese provinces (except Tibet, though the study did include one Tibetan county), representative of the adult population 45 years and older, in order to understand patterns of cataract surgery incidence across China between 2011 and 2015. The aim was to investigate incident cataract surgery, and to investigate the clinical (such as the presence of hypertension, diabetes, and depression), biological, and socio-economic determinants of and barriers to cataract surgery in adults aged 45 years and above in urban and rural China between 2011 and 2015.

Methods:

Study population

The China Health and Retirement Longitudinal Study (CHARLS) is a nationally-representative longitudinal survey among Chinese persons aged 45 years and older, that includes assessments of biological, social and economic conditions. We used data from the baseline study conducted between July and September 2011 ("Wave 1") and a follow up conducted (both surveys conducted in person) between July and September 2015 ("Wave 3"). Main respondents and spouses in the baseline survey are followed throughout the life of CHARLS, or until they die.[24] Detailed information on the methodology of CHARLS has been described elsewhere.[24] In brief, 17,708 people aged 45 years and older participated in the baseline study in 2011. Among them, 16,663 (94.1%) were eligible for data collection, 107 (0.604%) did not provide information on cataract surgery, 457 (2.58%) were aged

For peer review only - http://bmjopen.bmj.com/site/about/guidelines.xhtml

below 45 years, and 481 (2.72%) had already undergone cataract surgery before the baseline and were excluded from our sample. Among those eligible at baseline who provided information on cataract surgery in 2011 (n=16991, 96.0% of the total), 13705 (82.2%) attended the four-year follow-up in 2015. The protocols of the follow-up for the variables used in this paper followed those of the baseline study.

Samples were chosen through multistage probability sampling. In the first stage, 150 county-level units were randomly chosen with a probability-proportional-to-size (PPS) sampling technique from a sampling frame containing all county-level units with the exception of the Tibet Autonomous Region. The sample was stratified by region and within region by urban district or rural county and per capita statistics on gross domestic product (GDP). The final sample of 150 counties fell within 28 provinces. Our sample used the lowest level of government organization, consisting of administrative villages (*cun*) in rural areas and neighborhoods (*shequ* or *juweihui*) in urban areas, as primary sampling units (PSUs). We selected 3 PSUs within each county-level unit, using PPS sampling (more details are reported elsewhere).[24]

Variables

Cataract surgery incidence refers to the proportion of the cohort population aged 45 years and older who underwent cataract surgery in at least one eye between 2011 and 2015, among those who had not undergone cataract surgery in either eye at baseline. This was determined by participants' response to the question "Have you had cataract surgery before?", comparing answers between the previous and present round.

Age and gender were self-reported variables taken from the baseline 2011 survey. Distance and near vision were self-reported at baseline, using a scale of 1 to 5, with 1 being excellent and 4 and 5 being fair and poor respectively. Binary visual impairment was also self-reported.

For peer review only - http://bmjopen.bmj.com/site/about/guidelines.xhtml

Rural or urban place of residence was obtained from self-reported *Hukou*. *Hukou* is a Chinese internal passport system that is designed to regulate the influx of rural residents into urban areas, and migrants with rural *hukou* do not have equal access to health care compared to local urban residents. Hukou also limits the type of public health insurance the person has access to.

Three comorbidities linked with cataract and/or uptake of cataract surgery were extracted from CHARLS. Hypertension was defined as either self-report of a physician's diagnosis, or presence of objective biomarkers (systolic pressure >= 140 and/or diastolic pressure >= 90 mmHg) during direct examination. Diabetes was similarly defined by self-report of prior diagnosis by a doctor or the presence of a biomarker (HbA1c value ≥6.5%) on examination. Depression was measured using the Center for Epidemiologic Studies Depression Scale (CES-D-10)[25] with total score ranging from 0 (minimal symptoms) to 30 (most symptomatic).

Data were obtained from the National School of Development (China Centre for Economic Research) of Peking University. Secondary analysis of data from CHARLS did not require ethical approval. GDP data were extracted from the China Statistical Yearbook, and per capita household expenditure were derived from surveys of household expenditure divided by the number of household members. GDP in Chinese Yuan was converted to the US dollar using the exchange rate on 1st August, 2011, released by the center for foreign trade, Bank of China.

All data collected in the CHARLS are maintained at the National School of Development of Peking University, Beijing, China. The datasets are available from https://charls.charlsdata.com/pages/data/111/zh-cn.html.[26] The CHARLS was approved by the Ethical Review Committee of Peking University, and all participants gave written informed consent at the time of participation. This study used public data and therefore does not require additional ethics approval. The original CHARLS study from which the data were collected was carried out in Beijing and has obtained ethics approval from the Ethical Review Committee of Beijing University the ethical

For peer review only - http://bmjopen.bmj.com/site/about/guidelines.xhtml

approval number is IRB00001052-11015.

Patient and Public Involvement (PPI)

As this is an analysis of publicly available dataset, neither ethics nor PPI are applicable.

Statistical analysis

All statistical analyses were performed using Stata 13.0 (StataCorp., College Station, TX). The t-test was used to compare continuous variables, while Pearson X^2 or Fisher's exact tests were used for the comparison of categorical data. As a test to assess accuracy of household expenditure data from the sample as an index of income in the province, a scatterplot graph was made comparing household expenditure to provincial GFDP data. The 4-year incidence of cataract surgery stratified by age and gender were calculated. Age was defined in our analysis as the age at baseline. Univariate and multivariate logistic regression models were used to test for association with incident cataract surgery over the 4-year follow-up period. We ran three sets of regression analysis, one including the whole sample of eligible participants (i.e., all eligible people who were aged 45 years and above), one including those eligible participants aged 60 years and above (as visually-significant cataract is far more prevalent in this age range), and the third including those eligible participants aged 60 years and above who also reported fair or poor vision (the subset of participants at greatest risk for undergoing cataract surgery) . The variables in univariate models that had p value of less than 0.05 were deemed significant and were kept in the multivariate models. The incidence of cataract surgery in each province was affected by the age and gender structure of the population aged 45 years and over. Therefore, the provincial incidence of cataract surgery was adjusted for age and gender structure of the population. We applied sample weights to the cataract surgery incidence between 2011 and 2015. These weights were constructed from weights for the structure of age and gender from the baseline wave of the CHARLS study. A p value of <0.05 was defined as statistically significant.

For peer review only - http://bmjopen.bmj.com/site/about/guidelines.xhtml

Results:

Among the 16,663 eligible people aged 45 years and above (Table 1), 13,705 (82.2%) attended the year four follow-up. Among them, 13,538 (98.8%) did not report receiving surgery between 2011 and 2015, while 167 (1.22%) did. Those who received surgery were significantly older (66.17+/-8.79 vs 58.29+/-9.18, p=<0.001), and more likely to be illiterate (45% vs 27%, p<0.001), have self-reported poor distance vision at baseline (49.7% vs 20.0%, p=< 0.001), self-reported poor near vision at baseline (37.1% vs 21.8%, p=< 0.001), self-reported visual impairment at baseline (15.6% vs 5.5%, p=< 0.001), diabetes (12.0% vs 7.4% ,p=< 0.05), and higher depression scores at baseline (9.70 vs 8.37 on a scale of 0-30, p=< 0.05)

Table 1: Baseline characteristics of participants aged 45 and above who participated both the baseline and follow-up surveys.

**Baseline characteristic N=13705， >=45， drop missing cases**	**Cataract surgery<br>N=167**	**No cataract surgery<br>N=13538**
Age (years), mean (Std. Dev.)	66.17(8.79)	58.29(9.18)
Distance Vision status, n (%)		
Poor or fair	125 (75.9)	7782 (57)
Excellent, very good and good	32 (19.2)	4904 (36)
Hypertension, n (%)		
Yes	67 (40),	5238 (39)
No	76 (46)	6532 (48)
Diabetes, n (%)		
Yes	20 (12)	1004 (7.4)
No	102 (61)	8701 (64)

For peer review only - http://bmjopen.bmj.com/site/about/guidelines.xhtml

Male	71(42)	6556(48)
Female gender, n (%)	96(58)	6982(52)
Literacy, n (%)		
Literate	92(55)	9865(73)
Illiterate	75(45)	3673(27)
mean depression score, mean (SD)	9.70(6.53)	8.37(6.30)
Hukou, n (%)		
Rural	127（76.0）	10961（80）
Non-rural	39(23)	2484(18)
Log household per capita expenditure (USD), mean (SD)	4.57 (.94)	4.68(.88)
Health insurance, n (%)		
Yes	158 (95)	12664(94)
No	8 (5)	797(6)
Geographical location, n (%)		
Eastern region	52(31)	4704(35)
Central region	60(36)	4428(33)
Western region	55(33)	3306(34)
marital status		
married	131(78)	11066(82)
Not married	36 (22)	2472 (18)

Table 2 lists the 28 provinces for which data are available in the current study in order of per capita GDP by region and by province within region. There was a non-significant trend towards higher mean weekly household per

For peer review only - http://bmjopen.bmj.com/site/about/guidelines.xhtml

capita expenditure in Eastern China (US$34.3) versus Central (US$20.5) and Western China (US$18.1). Mean provincial per capita weekly household expenditures as reported by study participants were highly correlated ($R2=0.650$, $P=0.0018$, regression correlation) with available data for per capita GDP by province (Figure 1).

Table 2: Characteristics of 28 Chinese provinces with regard to per capita gross domestic product (GDP), population and other characteristics, in 2011 (Arranged by region and province in order of reported per capita GDP)

Province name	Reported per capita GDP （USD, 2011）	Reported total population (thousands) (2011)	Reported population Density (persons/ square kilometre) (2011)	Number of participants in the current study （N=16663）	Mean weekly household per capita expenditure of study participants (n=16,663), 2011 (USD)	Mean household per capita expenditure, 2011, by geographic region (USD)
Eastern China						
Tianjin	13232.0	13,550	2636	149	44.9	34.3
Beijing	12680.0	20,190	1428	85	73.8	
Shanghai	12820.1	23,470	3702	58	58.6	
Jiangsu	9672.5	78,990	2013	834	38.8	
Zhejiang	9200.3	54,630	1741	659	48.0	
Guangdong	7889.4	105,050	2637	913	31.9	
Liaoning	7882.1	43,830	1712	510	24.3	
Fujian	7356.8	37,200	2306	483	36.5	
Shandong	7350.3	96,370	1389	1374	24.4	
Jilin	5972.1	27,490	2371	415	32.6	
Hebei	5274.8	72,410	2362	712	24.1	
Heilongjiang	5096.2	38,340	5146	312	39.5	

For peer review only

For peer review only - http://bmjopen.bmj.com/site/about/guidelines.xhtml

TOTAL	8702.2	50,960	2454	6504	39.8	
Central China						
Hubei	5310.2	57,580	1969	518	27.7	
Shanxi	4869.2	35,930	2977	511	22.3	
Hunan	4639.8	65,960	2908	773	28.3	
Henan	4450.5	93,880	5124	1313	24.7	20.5
Jiangxi	4060.6	44,880	4527	815	29.7	
Anhui	3984.4	59,680	2265	816	23.4	
TOTAL	4552.5	59,652	3295	4746	26.0	
Western China						
Inner Mongolia	9002.3	24,820	764	819	44.3	
Chongqing	5357.2	29,190	1830	219	29.5	
Shaanxi	5196.4	37,430	5821	532	45.3	
Xinjiang	4672.0	22,090	4563	96	26.2	
Qinghai	4584.2	5680	2487	144	25.1	
Sichuan	4058.0	80,500	2782	1433	25.7	
Guangxi	3932.7	46450	1569	582	22.2	18.1
Gansu	3042.7	25640	3824	402	24.8	
Yunnan	2991.5	46310	3811	997	21.7	
Guizhou	2548.6	34690	3502	189	23.9	
TOTAL	4538.6	35,280	3095	5413	28.9	

Among 7052 (42.3%) participants in the current study aged 60 and above, 126 indicated they had undergone cataract surgery during 2011 to 2015. Among 4522 (27.1%) participants aged 60 years and above and reporting

For peer review only - http://bmjopen.bmj.com/site/about/guidelines.xhtml

For peer review only

"fair" or "poor" vision at the time of the most recent survey four years previously, data on cataract surgery were missing for 38 persons (0.840%), and a total of 98 (2.19% or 98/4484) indicated they had undergone cataract surgery, the 4-year incidence rate ranging from 2.43% (31/1401) in Central China, to 2.27% (35/1545) in the East to 2.08% (32/1538) in the West. (Table 3)

Table 3: Characteristics of persons aged 60 and over with self-reported vision of fair or poor in 2011 (N=4522) by region. Regions are listed in order of per capita GDP in 2011.

Economic region	Mean age (SD)	Women, n (%)	Literate, n (%)*	Mean depression score (95%CI) (SD)***	Rural (versus urban) residence, n (%)*	Mean household per capita expenditure, USD (SD)	Has only public health insurance, n (%)***	4-year cataract surgery incidence****, (%)
Eastern region	68.7 (0.181)	814 (52.7)	978 (63.3%)	9.18 （8.86-9.50） (0.164)	1,151 (74.6%)	24.7 (1.05)	1399 （90.6）	35/1,545 (2.27%)
Central region	67.8 (0.177)	705 (50.3%)	840 (60.0%)	10.1 （9.78-10.49） (0.179)	1,130 (80.7%)	19.7 (0.728)	1292 （92.2）	31/1,401 (2.43%)
Western region	68.4 (0.170)	791 (51.4%)	909 (59.1%)	10.9 （10.57-11.24） （0.17）	1,228 (79.8%)	21.2 (0.854)	1397 （90.8）	32/1,538 (2.08%)
TOTAL	68.34 (0.101)	2,327 (51.46%)	2,727/4484	10.07	3509/4482	21.8	4088/4422	98/4484 (2.19%)

For peer review only - http://bmjopen.bmj.com/site/about/guidelines.xhtml

			(60.82%)	(9.88-10.23) (.099)	(78.29%)	(0.5)	(92.45%)	

* P< 0.05, ** P < 0.01, *** P < 0.001, Chi square test for trend from highest to lowest cataract surgery incidence

****Data on cataract surgery was missing for 38 persons (0.840%)

For peer review only

In linear regression models of potential predictors of regional incidence of cataract surgery among persons aged 45 years and above at baseline, older age, worse vision, having hypertension or diabetes, being illiterate, and higher depression score were all significantly associated with increased surgery rates in the univariate model, and all these factors remained significant in multivariate model (Table 4). Results were similar in the multivariate regression model including only persons aged 60 years and above, except that urban hukou was also associated with higher incidence of surgery (Table 5). When only those aged 60 years and above with self-reported fair or poor vision were included, results in the multivariate model were again the same, except that higher household expenditure was also associated with higher incidence of surgery (Table 6).

Table 4: Linear regression model of potential predictors of the incidence of cataract surgery among all persons aged > =45 years (n= 16663, among whom 167 [1.00%] reported having surgery) across whole of China between 2011 and 2015 **(Bold type indicates values significant at P<0.05)**

Baseline Characteristic	Univariate model			Multivariate model		
	Beta coefficient	95% Confidence Interval	P-value	Beta coefficient	95% Confidence Interval	P-value
Age	**.094**	**-.0013 -.0009**	**<0.001**	**.080**	**.00098 .00106**	**<0.001**
Poor or Fair self-reported vision	**0.41**	**.005 .013**	**<0.001**	**.022**	**00485 .00625**	**<0.001**
Hypertension present	**.005**	**.001 .002**	**<0.001**	**-.018**	**-.0048 -.0035**	**<0.001**

For peer review only - http://bmjopen.bmj.com/site/about/guidelines.xhtml

(adjusted for age)						
Diabetes present (adjusted for age)	**.023**	**.008 .010**	**<0.001**	**.019**	**.0063 .0085**	**<0.001**
Female sex	.013	-.001 .007	0.129			
Literate	**-.0438**	**-.015 .007**	**<0.001**	**-.021**	**-.00614 -.00468**	**<0.001**
Mean depression score (Adjusted for age)	**.023**	**.0004 .0005**	**<0.001**	**.012**	**.00017 .00028**	**<0.001**
Rural (vs non-rural) hukou	-.014	-.0008 .0085	0.106			
Log household per capita expenditure	-.013	-.0038 .0006	0.146			
Reported having health insurance	.005	-.0054 .0102	0.550			

1
2

For peer review only - http://bmjopen.bmj.com/site/about/guidelines.xhtml

Table 5: Linear regression model of potential predictors of the regional incidence of cataract surgery among all persons aged > =60 years (n= 7052, among whom 126 [1.79%] reported having surgery) **(Bold type indicates values significant at P<0.05)**

Baseline Characteristic	Univariate model			Multivariate model*		
	Beta coefficient	95% Confidence Interval	P-value	Beta coefficient	95% Confidence Interval	P-value
Age	**0.060**	**.0007，.020**	**<0.001**	**.036**	**.00077 .00096**	**<0.001**
Poor or Fair self-reported vision	**.052**	**-.025 -.008**	**<0.001**	**.039**	**.01167 .01431**	**<0.001**
Hypertension present (adjusted for age)	**-.011**	**-.0043 -.0023**	**<0.001**	**-.023**	**-.00804 - .00567**	**<0.001**
Diabetes present (adjusted for age)	**.029**	**.0117 .0151**	**<0.001**	**.024**	**.00949 .01318**	**<0.001**
Female sex	.024	-.00005 .01499	0.068			
Literate	**-.032**	**-.0178 .0017**	**0.018**	**-.027**	**-.00957 - .00701**	**<0.001**
Mean depression score (Adjusted for age)	**.020**	**.000383 -.000536**	**<0.001**	**.023**	**.00044 .00062**	**<0.001**

For peer review only - http://bmjopen.bmj.com/site/about/guidelines.xhtml

Rural (vs non-rural) hukou	**-.030**	**-.0204 -.0015**	**0.024**	**-.043**	**-.01801 -.01489**	**<0.001**
Log household per capita expenditure	.011	-.00277 .00642	0.435			
Reported having health insurance	.001	-.016 .017	0.926			

For peer review only

Table 6: Linear regression model of potential predictors of the regional incidence of cataract surgery among all persons aged > =60 years and those who reported fair or poor vision (n= 4522) **(Bold type indicates values significant at P<0.05)**

Baseline Characteristic	Univariate model			Multivariate model*		
	Beta coefficient	95% Confidence Interval	P-value	Beta coefficient	95% Confidence Interval	P-value
Age	**.064**	**.0008227 .002492**	**<0.001**	**.035**	**.00076 .00101**	**<0.001**
Hypertension present (adjusted for age)	**-.014**	**-.00596 -.0032489**	**<0.001**	**-.039**	**-.01425 -.01100**	**<0.001**
Diabetes present (adjusted for age)	**.041**	**.0184 .0229**	**<0.001**	**.041**	**.01744 .02250**	**<0.001**
Female sex	.024	-.00278 .01839	0.148			
Literate	-.027	-.01995 .00171	0.099			
Mean depression score (Adjusted for age)	**.006**	**.0000485 .0002474**	**0.004**	**.027**	**.00053 .00078**	**0.004**
Rural (vs non-rural) hukou	**-.041**	**-.030558 -.003757**	**0.012**	**- .031**	**-.01515 -.01066**	**<0.001**

For peer review only - http://bmjopen.bmj.com/site/about/guidelines.xhtml

Log household per capita expenditure	**.036**	**.0001394 .012749**	**0.045**	**.012**	**.00130 .0032**	**<0.001**
Reported having health insurance	-.001	-.022986 .021785	0.958			

Discussion:

The current study found that between 2011 and 2015, the 4-year incidence of self-reported cataract surgery among people aged 45 and above was relatively low (1.22%) compared to earlier reports, which ranged from 2.9% (5-year incidence) in the Beijing Eye Study[10] to 4.4% (5-year incidence) in Liwan, southern China.[12] It is worth noting that the Beijing and Liwan studies reported 5-year incidence rates, whereas our study reported 4-year incidence rate. This discrepancy may partly explain why our incidence rate appears to be lower. However, due to study limitations, we were unable to extend the follow-up period to 5 years. It is important to note that our sample was a nationally representative randomised sample selected from all provinces of China, from both urban and rural regions in each province, while previous studies only reported findings from one specific urban region in a particular province. This difference in sample selection may also contribute to the variance in incidence rates. Moreover, in our study, cataract surgery was self-reported, whereas in other studies it was extracted from medical records or clinical examination. More data are needed to help policy makers address the barriers to uptake of cataract surgery in China.[19-22] Previous studies have examined institutional predictors of higher surgical capacity at the institutional level (principally screening outreach activities). This is first large population study of determinants of surgery uptake in China, providing complementary information.

For peer review only - http://bmjopen.bmj.com/site/about/guidelines.xhtml

Our finding of lower surgical rates in more rural provinces is in agreement with results from prior population studies[8,13] which have also reported especially low rates of surgical uptake in rural settings. This finding indicates that this lower rate persists despite the rapid expansion of the NCMS rural health insurance program.[27,28]

With respect to individual-level variables, it was unsurprising that worse self-reported vision, as captured by three different variables, was significantly associated with greater uptake of surgery. As expected, older age was associated with elevated uptake of surgery.

Diabetes is well-established in population studies to be associated with higher risk of lens opacity, and so our observed higher prevalence of diabetes among those accepting surgery is not surprising. Additionally, cataract has been linked to higher rates of mental health problems, including depression,[29] which we believe could account for the observed higher rates of depression among those undergoing incident cataract surgery. To our knowledge, there is no evidence suggesting that depression drugs cause cataract. We conducted a thorough search of PubMed and were unable to find any studies supporting a causal link between depression drugs and cataract formation. A noteworthy individual-level finding was a higher prevalence of self-reported illiteracy among those who underwent incident cataract surgery. Previous population studies have consistently linked poor education and lack of knowledge about cataract with low surgical uptake.[22-23] One possible explanation for this finding is that illiterate people were more likely to comply with the doctor's recommendations. Conversely, those with higher levels of education may have been more cautious about using local services and may have been unable to afford distant care. Additionally, those with lower literacy levels may have other unmeasured factors that predispose them to higher rates of cataract, such as greater exposure to UV light. It is also possible that illiterate individuals provided inaccurate information regarding their cataract surgery or had other eye related procedures, which resulted in relatively unreliable data concerning cataract surgery.

For peer review only

Higher economic status (represented by higher weekly household per capita expenditure, which correlated strongly with per capita GDP, Fig 1) was associated with receiving cataract surgery among people aged 60 and above with poor vision. This result is expected, however, there have been several efforts in recent years that help provide surgery to those economically more disadvantaged. One is the roll-out of projects providing high-volume free surgery, such as the Million People Cataract Surgery Plan,[30] during the period covered in the current study. This has been supplemented by non-governmental organization (NGO)-supported cataract surgical programs by entities such as Lifeline Express,[31] and by the high penetration of the NCMS rural health insurance system, now covering some 98.9% of rural-dwellers[27] and substantially reducing out-of-pocket expenses associated with cataract surgery. We observed that the percentages of people covered by public health insurance did not vary significantly between poorer and richer regions. These programs taken together may have improved access in poor areas that reduced inequality in cataract surgery access associated with income, but in the end, those with greater income were more likely to have cataract surgery.

The strengths of this study include a large nationally representative sample from most provinces of China, selected using standardized protocols. Study personnel were trained in standard fashion to ensure all procedures were carried out as described. Our findings provide a basis for comparison with other low- and middle-income countries and especially those undergoing rapid economic transitions. Limitations must be acknowledged as well. In the first place, the study did not measure objective visual acuity due to time and cost. Our main outcome, having undergone cataract surgery, depended on patient report for similar reasons, though evidence has suggested that self-reported cataract surgery is accurate and reliable.[32] Finally, the results may be influenced by patients (17.8%) who did not come for the follow-up examination.

Conclusion:

For peer review only - http://bmjopen.bmj.com/site/about/guidelines.xhtml

Our study provides unique data on cataract surgical incidence in China. Our results suggest that surgical rates remain low. Rural and metropolitan both have access to cataract surgery, but underserved groups such as rural dwellers are less likely to receive cataract surgery.

Acknowledgement:
The authors would like to thank Professor Zhao Yaohui for her valuable comments and suggestions on this manuscript. We would also like to thank the CHARLS team for the data that enabled these analyses.

Author Contribution
CJ conceptualized the study, did a literature review, participated in data collection and data analysis, drafted the manuscript, and is responsible for the overall content as a guarantor. NC conceptualized the study, interpreted data, and revised the manuscript. XJ participated in data collection and data analysis, interpreted data, created the figure, and revised the manuscript. DF, YD, MH, RC, TB and LK participated in data interpretation and manuscript revision. All authors read and approved the final draft.

Competing interests: The authors have no competing interest.

Funding: N/A

Data sharing statement: All data collected in the CHARLS are maintained at the National School of Development of Peking University, Beijing, China. The datasets are available from http://charls.pku.edu.cn/pages/data/111/zh-cn. html.

Ethics approval statement: The CHARLS was approved by the Ethical Review Committee of Peking University, and all participants gave written informed consent at the time of participation. This study used public data and therefore does not require additional ethics approval. The original CHARLS study from which the data

For peer review only - http://bmjopen.bmj.com/site/about/guidelines.xhtml

were collected was carried out in Beijing and has obtained ethics approval from the Ethical Review Committee of Beijing University the ethical approval number is IRB00001052-11015.

For peer review only

For peer review only - http://bmjopen.bmj.com/site/about/guidelines.xhtml

References

1. Enoch J, McDonald L, Jones L, Jones PR, Crabb DP. Evaluating whether sight is the most valued sense. *JAMA Ophthalmol* 2019; **137**(11): 1317-20.

2. Bourne RR, Stevens GA, White RA, et al. Causes of vision loss worldwide, 1990-2010: a systematic analysis. *Lancet Glob Health* 2013; **1**(6): e339-49.

3. Liang YB, Friedman DS, Wong TY, et al. Prevalence and causes of low vision and blindness in a rural Chinese adult population: the Handan Eye Study. *Ophthalmology* 2008; **115**(11): 1965-72.

4. Horton S, Gelband H, Jamison D, Levin C, Nugent R, Watkins D. Ranking 93 health interventions for low- and middle-income countries by cost-effectiveness. *PLoS One* 2017; **12**(8).

5. Lee BS, Munoz BE, West SK, Gower EW. Functional improvement after one-and two-eye cataract surgery in the Salisbury Eye Evaluation. *Ophthalmology* 2013; **120**(5): 949-55.

6. Wang JJ, Mitchell P, Smith W. Vision and low self-rated health: the Blue Mountains Eye Study. *Invest Ophthalmol Vis Sci*. 2000; **41**(1): 49-54.

7. Lopreite M, Zhu Z. The effects of ageing population on health expenditure and economic growth in China: A Bayesian-VAR approach. Social science & medicine. 2020 Nov 1;265:113513.

8. Zhao J, Ellwein LB, Cui H, et al. Prevalence and outcomes of cataract surgery in rural China: the China nine-province survey. *Ophthalmology* 2010; **117**(11): 2120-8.

9. Lavanya R, Wong TY, Aung T, et al. Prevalence of cataract surgery and post-surgical visual outcomes in an urban Asian population: the Singapore Malay Eye Study. *Br J Ophthalmol*. 2009; **93**(3): 299-304.

10. Liu B, Xu L, Wang YX, Jonas JB. Prevalence of cataract surgery and postoperative visual outcome in Greater Beijing: the Beijing Eye Study. *Ophthalmology* 2009; **116**(7): 1322-31.

11. Li EY, Liu Y, Zhan X, et al. Prevalence of blindness and outcomes of cataract surgery in Hainan Province in South China. *Ophthalmology* 2013; **120**(11): 2176-83.

For peer review only - http://bmjopen.bmj.com/site/about/guidelines.xhtml

12. Huang W, Huang G, Wang D, Yin Q, Foster PJ, He M. Outcomes of cataract surgery in urban southern China: the Liwan Eye Study. *Invest Ophthalmol Vis Sci*. 2011; **52**(1): 16-20.

13. Zhao J, Xu X, Ellwein LB, et al. Cataract Surgical Coverage and Visual Acuity Outcomes in Rural China in 2014 and Comparisons With the 2006 China Nine-Province Survey. *Am J Ophthalmol* 2018; **193**: 62-70.

14. Zhang JS, Xu L, Wang YX, You QS, Da Wang J, Jonas JB. Five-year incidence of age-related cataract and cataract surgery in the adult population of greater Beijing: the Beijing Eye Study. *Ophthalmology* 2011; **118**(4): 711-8.

15. Huang W, Zheng Y, Wang L, et al. Five-year incidence and postoperative visual outcome of cataract surgery in urban southern China: the Liwan Eye Study *Invest Ophthalmol Vis Sci*. 2012; **53**(13): 7936-42.

16. Kanthan GL, Wang JJ, Rochtchina E, et al. Ten-year incidence of age-related cataract and cataract surgery in an older Australian population: the Blue Mountains Eye Study. *Ophthalmology* 2008; **115**(5): 808-14.

17. Klein BEK, Klein R, Lee KE. Incidence of age-related cataract over a 10-year interval: the Beaver Dam Eye Study. *Ophthalmology* 2002; **109**(11): 2052-7.

18. Koo E, Chang JR, Agrón E, et al. Ten-year incidence rates of age-related cataract in the Age-Related Eye Disease Study (AREDS): AREDS report no. 33. *Ophthalmic Epidemiol* 2013; **20**(2): 71-81.

19. Li Z, Song Z, Wu S, et al. Outcomes and barriers to uptake of cataract surgery in rural northern China: the Heilongjiang Eye Study. *Ophthalmic Epidemiol* 2014; **21**(3): 161-8.

20. Zhang M, Wu J, Li L, et al. Impact of cataract screening outreach in rural China. *Invest Ophthalmol Vis Sci* 2010; **51**(1): 110-4.

21. Yin Q, Hu A, Liang Y, et al. A two-site, population-based study of barriers to cataract surgery in rural China. *Invest Ophthalmol Vis Sci*. 2009; **50**(3): 1069-75.

22. Zhang M, Wu X, Li L, et al. Understanding barriers to cataract surgery among older persons in rural China through focus groups. *Ophthalmic Epidemiol* 2011; **18**(4): 179-86.

For peer review only - http://bmjopen.bmj.com/site/about/guidelines.xhtml

23. An L, Jan CL, Feng J, Wang Z, Zhan L, Xu X. Inequity in access: cataract surgery throughput of Chinese ophthalmologists from the China national eye care capacity and resource survey. Ophthalmic Epidemiology. 2020 Jan 2;27(1):29-38.

24. Zhao Y, Hu Y, Smith JP, Strauss J, Yang G. Cohort profile: the China health and retirement longitudinal study (CHARLS). *Int J Epidemiol*. 2014; **43**(1): 61-8.

25. Lewinsohn PM, Seeley JR, Roberts RE, Allen NB. Center for Epidemiologic Studies Depression Scale (CES-D) as a screening instrument for depression among community-residing older adults. *Psychol Aging*. 1997 Jun;12(2):277.

26. (Dataset) https://charls.charlsdata.com/pages/data/111/zh-cn.html (Last accessed 3 Feb 2023)

27. Gu D, Yang X, Li X, Liang C, Zhong J, Feng N. Innovating new rural cooperative medical scheme (NCMS) for better patient satisfaction in rural China. Int J Env Res Pub Health. 2018 Sep;15(9):2007.

28. Drinkwater JJ, Davis WA, Davis TM. A systematic review of risk factors for cataract in type 2 diabetes. Diabetes/metabolism research and reviews. 2019 Jan;35(1):e3073.

29. Pellegrini M, Bernabei F, Schiavi C, Giannaccare G. Impact of cataract surgery on depression and cognitive function: Systematic review and meta-analysis. Clin Exper Ophthalmol. 2020 Mar 27.

30. Yan X, Guan C, Mueller A, Iezzi B, He M, Liang H, Meltzer M, Congdon NG. Outcomes and predicted impact on vision restoration of the China Million Cataract Surgeries Program. *Ophthalmic Epidemiol*. 2013;20:294-300.

31. Lifeline Express home page. http://www.lifeline-express.com/. Last visited 4 December 2020.

32. Bowie H, Congdon N, Lai H, West SK. Validity of a personal and family history of cataract and cataract surgery in genetic studies. *Invest Ophthalmol Clin Sci* 2003;44:2905-8.

For peer review only - http://bmjopen.bmj.com/site/about/guidelines.xhtml

Figure 1: Scatterplot and regression line showing the reported per capita gross domestic product of provinces (USD) against the mean household per capita expenditure (USD) of participants in our study from these provinces.

For peer review only

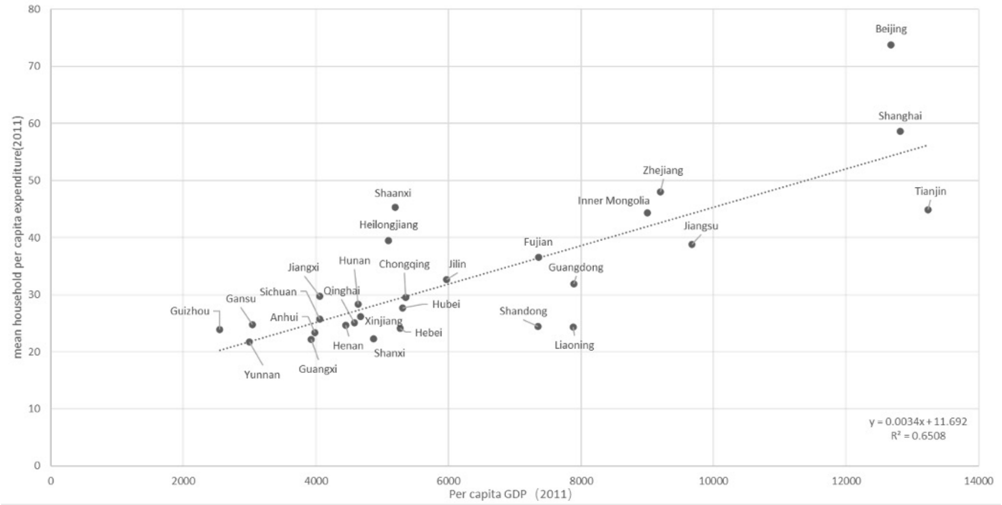

184x93mm (144 x 144 DPI)

STROBE Statement—checklist of items that should be included in reports of observational studies

	Item No.	Recommendation	Page No.	Relevant text from manuscript
**Title and abstract**	1	(*a*) Indicate the study's design with a commonly used term in the title or the abstract	Page 1 lines 1-2	
		(*b*) Provide in the abstract an informative and balanced summary of what was done and what was found	Page 3 lines 1-33, page 4 lines 1-7	
**Introduction**				
Background/rationale	2	Explain the scientific background and rationale for the investigation being reported	Page 5 lines 1-24, page 6 lines 1-6	
Objectives	3	State specific objectives, including any prespecified hypotheses	Page 6 lines 8-13	
**Methods**				
Study design	4	Present key elements of study design early in the paper	Page 6 line 15-24, page 7 lines 1-13	
Setting	5	Describe the setting, locations, and relevant dates, including periods of recruitment, exposure, follow-up, and data collection	Page 7 lines 2-13	
Participants	6	(*a*) *Cohort study*—Give the eligibility criteria, and the sources and methods of selection of participants. Describe methods of follow-up<br>*Case-control study*—Give the eligibility criteria, and the sources and methods of case ascertainment and control selection. Give the rationale for the choice of cases and controls<br>*Cross-sectional study*—Give the eligibility criteria, and the sources and methods of selection of participants	Page 6 lines 22-24, page 7 lines 1-2	
		(*b*) *Cohort study*—For matched studies, give matching criteria and number of exposed and unexposed<br>*Case-control study*—For matched studies, give matching criteria and the number of controls per case	NA	
Variables	7	Clearly define all outcomes, exposures, predictors, potential confounders, and effect modifiers. Give diagnostic criteria, if applicable	Page 7 lines 15-22, page 8 lines 1-17	
Data sources/ measurement	8*	For each variable of interest, give sources of data and details of methods of assessment (measurement). Describe comparability of assessment methods if there is more than one group	Page 7 lines 15-22, page 8 lines 1-17	
Bias	9	Describe any efforts to address potential sources of bias	NA	

For peer review only - http://bmjopen.bmj.com/site/about/guidelines.xhtml

Study size	10	Explain how the study size was arrived at	Page 7 lines 6-13
Quantitative variables	11	Explain how quantitative variables were handled in the analyses. If applicable, describe which groupings were chosen and why	Page 9 lines 7-23
Statistical methods	12	(*a*) Describe all statistical methods, including those used to control for confounding	Page 9 lines 7-23
		(*b*) Describe any methods used to examine subgroups and interactions	NA
		(*c*) Explain how missing data were addressed	NA
		(*d*) *Cohort study*—If applicable, explain how loss to follow-up was addressed<br>*Case-control study*—If applicable, explain how matching of cases and controls was addressed<br>*Cross-sectional study*—If applicable, describe analytical methods taking account of sampling strategy	NA
		(*e*) Describe any sensitivity analyses	NA
**Results**			
Participants	13*	(a) Report numbers of individuals at each stage of study—eg numbers potentially eligible, examined for eligibility, confirmed eligible, included in the study, completing follow-up, and analysed	Page 10 lines 1-8
		(b) Give reasons for non-participation at each stage	NA
		(c) Consider use of a flow diagram	NA
Descriptive data	14*	(a) Give characteristics of study participants (eg demographic, clinical, social) and information on exposures and potential confounders	Page 10 lines 4-8
		(b) Indicate number of participants with missing data for each variable of interest	NA
		(c) *Cohort study*—Summarise follow-up time (eg, average and total amount)	Page 10 lines 2-3
Outcome data	15*	*Cohort study*—Report numbers of outcome events or summary measures over time	Page 10 lines 2-3
		Case-control study—Report numbers in each exposure category, or summary measures of exposure	
		Cross-sectional study—Report numbers of outcome events or summary measures	
Main results	16	(*a*) Give unadjusted estimates and, if applicable, confounder-adjusted estimates and their precision (eg, 95% confidence interval). Make clear which confounders were adjusted for and why they were included	Pages 10-21
		(*b*) Report category boundaries when continuous variables were categorized	Pages 10-21
		(*c*) If relevant, consider translating estimates of relative risk into absolute risk for a meaningful time period	NA

Continued on next page

For peer review only - http://bmjopen.bmj.com/site/about/guidelines.xhtml

Other analyses	17	Report other analyses done—eg analyses of subgroups and interactions, and sensitivity analyses	NA
**Discussion**			
Key results	18	Summarise key results with reference to study objectives	Page 21 lines 5-15, page 22 lines 1-24, page 23 lines 1-3
Limitations	19	Discuss limitations of the study, taking into account sources of potential bias or imprecision. Discuss both direction and magnitude of any potential bias	Page 23 lines 8-12
Interpretation	20	Give a cautious overall interpretation of results considering objectives, limitations, multiplicity of analyses, results from similar studies, and other relevant evidence	Page 21 lines 5-15, page 22 lines 1-24, page 23 lines 1-3
Generalisability	21	Discuss the generalisability (external validity) of the study results	NA
**Other information**			
Funding	22	Give the source of funding and the role of the funders for the present study and, if applicable, for the original study on which the present article is based	NA

*Give information separately for cases and controls in case-control studies and, if applicable, for exposed and unexposed groups in cohort and cross-sectional studies.

Note: An Explanation and Elaboration article discusses each checklist item and gives methodological background and published examples of transparent reporting. The STROBE checklist is best used in conjunction with this article (freely available on the Web sites of PLoS Medicine at http://www.plosmedicine.org/, Annals of Internal Medicine at http://www.annals.org/, and Epidemiology at http://www.epidem.com/). Information on the STROBE Initiative is available at www.strobe-statement.org.

For peer review only
